# The Chemokines Initiating and Maintaining Immune Hot Phenotype Are Prognostic in ICB of HNSCC

**DOI:** 10.3389/fgene.2022.820065

**Published:** 2022-05-27

**Authors:** Yuhong Huang, Han Liu, Xuena Liu, Nan Li, Han Bai, Chenyang Guo, Tian Xu, Lei Zhu, Chao Liu, Jing Xiao

**Affiliations:** ^1^ Department of Oral Pathology, School of Stomatology, Dalian Medical University, Dalian, China; ^2^ Dalian Key Laboratory of Basic Research in Oral Medicine, School of Stomatology, Dalian Medical University, Dalian, China; ^3^ Department of Nuclear Medicine, The 2nd Hospital Affiliated to Dalian Medical University, Dalian, China

**Keywords:** squamous cell carcinoma of head and neck (HNSCC), tumor-associated monocyte/macrophage (TAMM), immune checkpoint blockade (ICB), tumor micro-environment (TME), CXCL, CCL5, PD-1/PD-L1

## Abstract

**Background:** The immune checkpoint blockade (ICB) with anti-programmed cell death protein 1(PD-1) on HNSCC is not as effective as on other tumors. In this study, we try to find out the key factors in the heterogeneous tumor-associated monocyte/macrophage (TAMM) that could regulate immune responses and predict the validity of ICB on HNSCC.

**Experimental Design:** To explore the correlation of the TAMM heterogeneity with the immune properties and prognosis of HNSCC, we established the differentiation trajectory of TAMM by analyzing the single-cell RNA-seq data of HNSCC, by which the HNSCC patients were divided into different sub-populations. Then, we exploited the topology of the network to screen out the genes critical for immune hot phenotype of HNSCC, as well as their roles in TAMM differentiation, tumor immune cycle, and progression. Finally, these key genes were used to construct a neural net model via deep-learning framework to predict the validity of treatment with anti-PD-1/PDL-1

**Results:** According to the differentiation trajectory, the genes involved in TAMM differentiation were categorized into early and later groups. Then, the early group genes divided the HNSCC patients into sub-populations with more detailed immune properties. Through network topology, CXCL9, 10, 11, and CLL5 related to TAMM differentiation in the TME were identified as the key genes initiating and maintaining the immune hot phenotype in HNSCC by remarkably strengthening immune responses and infiltration. Genome wide, CASP8 mutations were found to be key to triggering immune responses in the immune hot phenotype. On the other hand, in the immune cold phenotype, the evident changes in CNV resulted in immune evasion by disrupting immune balance. Finally, based on the framework of CXCL9-11, CLL5, CD8^+^, CD4^+^ T cells, and Macrophage M1, the neural network model could predict the validity of PD-1/PDL-1 therapy with 75% of AUC in the test cohort.

**Conclusion:** We concluded that the CXCL9, 10,11, and CCL5 mediated TAMM differentiation and constructed immune hot phenotype of HNSCC. Since they positively regulated immune cells and immune cycle in HNSCC, the CXCL9-11 and CCL5 could be used to predict the effects of anti-PD-1/PDL-1 therapy on HNSCC.

## Introduction

Application of immune checkpoint blockage (ICB) has significantly improved the prognosis of multiple tumors, but exerted limited effects on head and neck squamous cell carcinoma (HNSCC) because less than 30% of the patients got a better prognosis (Curran et al., 2010; Topalian et al., 2012; Seiwert et al., 2016; Clarke et al., 2021; García Campelo et al., 2021). To find out the HNSCC sub-populations susceptible to ICB therapy, various criteria have been proposed for HNSCC classification, by which HNSCC was classified into the enhanced and decreased immune subtypes with the immune-related genes (Cao et al., 2018), into the CD8^+^ high and CD8^+^ low subtypes with the density of infiltrating CD8^+^ T cells (Saloura et al., 2019), into the basal, mesenchymal, atypical, and classical subtypes with the integrated genomic characteristics (Walter et al., 2013), and even into the m6A^high^ and m6A^low^ subtypes with the N6-methyladenosine (m6A) methylation levels on mRNAs (Yi et al., 2020). Although these criteria explicated the clinical and immune characteristics of HNSCC from different perspectives, how these HNSCC subtypes formed and the roles of the genes involved in it remained elusive.

When inflammation took place, the tumor-associated monocyte/macrophages in circulating blood were motivated into the inflammatory focus to maintain homeostasis, eliminate pathogens, and balance immune responses (Shi & Pamer, 2011). There were three types of tumor associated monocyte/macrophages, namely, the classical (CD14^+^; CD16^−^), the non-classical (CD16^+^), and the intermediate tumor associated monocyte/macrophages (CD14^+^; CD16^+^) (Ziegler-Heitbrock et al., 2010). During tumorigenesis, the tumor-associated monocyte/macrophages in circulation (mainly the classical type) were chemoattracted into tumor focus, and differentiated gradually into dendritic cells (DC) and Tumor Associated Macrophages (TAM) (Movahedi et al., 2010; Franklin et al., 2014; Guilliams et al., 2014; Li B et al., 2020). More than 50% of the immune cells in tumor micro-environment (TME) were TAM that affected the migration, invasion, angiogenesis, and drug-resistance of tumors (Watters et al., 2005; Kimura et al., 2007; Zheng et al., 2018). The M1-like phenotype of TAM exhibited the inhibitory effects on tumors, such as the promoted inflammatory response and chemoattraction of immune cells (Goswami et al., 2017). Reversely, M2-like phenotype of TAM suppressed inflammatory response, and enhanced immune evasion, angiogenesis, and metastasis, which resulted in a poor prognosis (Hu et al., 2016; Seminerio et al., 2018). However, recent studies reported that M1-like phenotype of TAM was also related to the poor prognosis in HNSCC and medulloblastoma by suppressing inflammatory response and promoting metastasis (Lee et al., 2018; Xiao et al., 2018). Previously, TAM was thought mainly to be macrophage M2, while the increasing evidence indicated that TAM also exhibited the phenotype of macrophage M1, suggesting that TAM contained the third population of macrophages other than macrophage M1 and M2. The third macrophage population was supposed to co-express the M1 and M2 characteristics and transform into M1 or M2 in certain instances (Estko et al., 2015; L. ; Gao et al., 2016; Kloepper et al., 2016). All the above findings indicated that the role of TAM in tumor progression could be complicated and not simply attributed to macrophage M1 and M2. Since the TAM was differentiated from the tumor-associated monocyte/macrophages gradually, the differentiating and differentiated TAM were termed as tumor-associated monocyte/macrophages/Macrophages (TAMM) in recent studies (Cassetta et al., 2019; Singhal et al., 2019). More and more studies implicated the TAMM as the potential target of ICB therapy. The relevance between TAMM responses and ICB therapy has been established by bioinformatic methods. In triple negative breast cancer, machine learning identified the TAMM-expressed genes which were highly associated with the prognosis and ICB therapy, and constructed a model predicting the response to ICB therapy with the 100% validation queue AUC (Bao et al., 2021). Moreover, WGCNA was used to find that the marker genes expressed in TAMM of glioblastoma, which were highly correlated with prognosis and ICB therapy, and were more active in the patients susceptible to ICB therapy (Zhang et al., 2021). Despite this, there are relatively few studies on HNSCC concerning the role of TAMM in immune response and ICB therapy. Since TAMM differentiation endowed TAMM with heterogeneity dynamically, instead of statically, we proposed a criterion that combined the genes involved in TAMM differentiation with the immune cells to depict the immune phenotype and prognosis of HNSCC in more detail.

## Materials and Methods

### Data Collection

The single cell RNA-seq (scRNA-seq) data of GSE139324 (10X genomics), including the tumor infiltrating immune cells from 16 HPV negative patients and the immune cells from the peripheral blood of a healthy donor, and GSE103322 (Smart-seq2), containing 5,902 single cells from 18 HNSCC patients, were obtained from Gene Expression Omnibus (GEO). Multiomics data and clinical data of 502 HNSCC patients obtained from The Cancer Genome Atlas (TCGA) database (Supplementary Table S1), including mRNA expression (level 3, Illumina RNA-Seq), miRNA expression (level 3, Illumina miRNA-Seq), somatic copy number variation (CNV level 3, Affymetrix SNP 6.0), and somatic mutation (level 4, MAF files), were obtained from UCSC Xena browser. The array data and clinical data of five HNSCC cohorts, GSE65858 (n = 270), GSE40774 (n = 134), GSE39366 (n = 138), GSE117973 (n = 77), and GSE41613 (N = 97), were obtained from Gene Expression Omnibus (GEO) database (Supplementary Table S1). The bulk transcriptome data and clinical data of six cohorts accepted the PDL-1/PD-L1 antibody immunotherapy, namely GSE93157 (n = 65, Non-Small Cell Lung Carcinoma, HNSCC and Melanoma), GSE154538 (n = 8, gastrointestinal cancer), GSE141119 (n = 12, melanoma), GSE91061 (n = 109, melanoma and non-small cell lung cancer), GSE78220 (n = 28, melanomas), GSE176307 (n = 88, Metastatic Urothelial Cancer), and the IMvigor210 (n = 348, bladder cancer), were obtained from Gene Expression Omnibus (GEO) and the IMvigor210 database (Supplementary Table S2). GSE93157 was array data, while GSE154538, GSE141119, GSE91061, GSE78220, GSE176307, and the IMvigor210 were bulk transcriptome data. The mRNA-seq data from the HNSCC cell line that accepted the treatment of anti-tumor drugs were obtained from Genomics of Drug Sensitivity in Cancer (GDSC).

### Data Processing

In the scRNA-seq from GSE139324, with the exclusion of the genes detected in fewer than three cells, the cells containing mRNA more than 4,500 or less than 200, and the cells expressing mitochondria genes more than 10% transcripts, there were 19,718 genes from 39,711 qualified cells of total 39,994 cells (283 cells were screened out). Through SCT in seurant package SCT, the data from the 18 patients in GSE139324 cohort were integrated to screen out the non-biological inferences, such as batch effect. Similarly, in the scRNA-seq of GSE103322 (Smart2-seq without screen), there were 21,519 genes and 5,844 qualified cells from 18 patients integrated by SCT. The HNSCC RNA-seq counts [log_2_(rawcounts+1)] obtained from USCS through exp[log_2_(rawcounts+1)-1] were restored to raw counts, and then the log2(fpkm-uq+1) from USCS was used to compare them with the data from other databases. There were 501 HNSCC samples (one normal sample was excluded) for the subsequent analyses. The GSE65858, GSE40774, GSE39366, GSE117973, and GSE41613 were normalized prior to following analyses. There were 501 samples in GSE93157, GSE154538, GSE141119, GSE91061, GSE78220, GSE176307, and the IMvigor210 for the following analyses except defective and reiterated data. The data from the RNA-seq of GDSC2 cell line were normalized with TPM for subsequent processing.

### Analyses on Squamous Cell Carcinoma of Head and Neck scRNA-Seq Data

For the GSE139324 cohort: 1) the data integrated with “SCT” Seurat package was applied for PCA analysis to find out the first 50 principal component analysis (PCA); 2) Umap (Uniform Manifold Approximation and Projection for Dimension Reduction) dimension reduction was performed on the 50 PCAs. In this unsupervised clustering, the function of FindNeighbors in Seurat package was used to construct a KNN graph based on the Euclidean distance in PCA space (top 50 PCAs, k = 20), and then, the function of FindClusters **(**Louvain algorithm**)** was used to cluster the cells with the resolution of 0.1. The K-NN clustering classified the consequences undergoing the dimension reduction into four clusters, which were annotated by SingleR as NK cells (n = 14,925), T cells (n = 14,073), B cells (n = 2,789), and tumor-associated monocyte/macrophages cells (n = 7,924). 3) Tumor-associated monocyte/macrophages cells were classified by K-NN into seven further clusters. Cluster 0, 1, 2, and 4 were annotated by SingleR as tumor-associated monocyte/macrophages (n = 6,875), while the cluster3 (n = 312), 5 (n = 478), and 6 (n = 259) as T and B cells. 4) The T cells were applied for Multimodal reference mapping (Hao et al., 2021) and divided into eight clusters, namely, the CD4 CTL, CD4 Navie cells, CD4 TCM, CD4 TEM, CD8 Navie cells, CD8 TCM, CD8 TEM, and proliferating T cells (Supplementary Table S3). 5) The tumor-associated monocyte/macrophages were applied for GSVA analysis for function enrichment. 6) The tumor-associated monocyte/macrophages were applied for pseudotime analysis through Monocle2 package and Destiny package, which adopted different manners to reduce the dimensions of the high-dimensional data. The single cell was separated and projected into low-dimensional space to form a differentiation trajectory with knots. Each knot represented a similar status of differentiation. (1) Through the data of single cell lineage, Monocle 2 adopted the embedding converse diagraph to learn the explicit principal graph (Packer et al., 2019). 2) Destiny adopted the diffuse maps (differentiating cells follow noisy diffusion-like dynamics) to mimic the division from multipotent cells (Coifman et al., 2005). 7) The “InferCNV” R package 1.10.1 (Patel et al., 2014) and CellPhoneDB (Python edition) (Efremova et al., 2020) were performed on all clusters for CNV analysis (normal blood cells as control) and cell communication analysis. ${\text{CNV}}_{\text{k}}\left(\text{i}\right)=\overset{\text{i}+50}{\underset{\text{j}=\text{i}-50}{\sum }}{\text{E}}_{\text{k}}\left({\text{O}}_{\text{j}}\right)/101$ , where CNV(i) was the estimated relative copy number, and $E_{k}\left(O_{j}\right)$ was mRNA level, of the i^th^ gene in the cell k at the whole genomic scope. 8) The differentially expressed genes (DEG) between tumor-associated monocyte/macrophages C1 and C0 were summarized with the “Findmarker” Seurant package. Setting |log_2_fold Change|>1.3 and FDR<0.05 as the cutoff criteria, the Log_2_Fold Change >1.3 was regarded as the characteristics of the genes for the early differentiation of TAMM, while Log_2_FoldChange<-1.3 as the characteristics of the genes for the late differentiation of TAMM (Supplementary Table S4). For GSE103322 cohort: 1) PCA analysis was performed on the data integrated by “SCT” Seurat package. According to the specific markers, the cells were divided into the malignant epithelial cells (KRT14, KRT6A, EPCAM, n = 1939), Cancer associated fibroblasts (FAP, PDRN, n = 1,697), T cells (CD2, CD3D, n = 1,633), B cells (SLAMF7, CD79A n = 354), endothelial cells (PECAM1, VWF n = 75), and mono-macrophage cells (CD14, CD163, CD68, n = 146). 2) T cells were further classified with Multimodal reference mapping into eight clusters of CD4 CTL, CD4 Navie cells, CD4 TCM, CD4 TEM, CD8 Navie cells, CD8 TCM, CD8 TEM, and proliferating T cells (Supplementary Table S5).

### CIBERSORT and ESTIMATE for Immune Cell and Stromal Scores

For the one TCGA HNSCC and five GEO HNSCC cohorts, “CIBERSORT” and “ESTIMATE” R package were applied to calculate the contents of the 22 kinds of immune cells (1,000 permutations) and immune and stromal score.

### The Unsupervised Clustering on TCGA Squamous Cell Carcinoma of Head and Neck and GSE65858 Cohorts

According to the scores of the genes in the early TAMM differentiation and the 22 kinds of immune cells, the unsupervised clustering (through “ConsensuClusterPlus” R package) was applied to the samples with Euclidean distance and Ward (unsquared distances) linkage to get the sub-populations with different immune phenotype and prognosis.

### Associations of TCGA Squamous Cell Carcinoma of Head and Neck Subtype With DNA Methylation, CNVs and Mutations

The data of methylation probes were normalized with “wateRmelon” R package, and the difference in methylation probes were analyzed with “limma” R package. The evidently altered regions in genome were screened with GISTIC2.0. The numeric focal CNV values larger than 0.2 meant gain, while less than 0.2 meant loss. Through Somatic mutation data, the TMB (the number of non-synonymous mutations in every million bases of somatic cells) of each patient in the TCGA HNSCC subtype were calculated.

### Search for the DEGs in the Subtype of TCGA HNSCS and GSE65858 Cohorts

The DEGs were obtained by comparing the A3 to A1 subtype, and the A3 to B subtype in TCGA HNSCC and GSE65858 cohorts with “limma” in R package. The TCGA HNSCC cohorts were produced by RNA-seq, while the GSE65858 resulted from micro-array. One criterion failed to satisfy the cohorts from a different sequencing approach. If the threshold of GSE65858 was identical to that for TCGA HNSC, the DEGs would be rare. For the TCGA HNSCC cohort, |Log_2_Fold Change|>1 and FDR<0.05 were set up as standard. According to the DEGs in GSE65858 array, a threshold of |Log_2_Fold Change|>0.2 and FDR<0.05 was selected to keep the numbers of DEGs in the two cohorts from varying too much. FDR was the *p* value calibrated using the Benjamini–Hochberg method.

### Confirmation of the Key Genes

In the two HNSCC cohorts, the comparison between A3 and B subtypes gave rise to 181 overlapped candidate genes. Centiscape was applied to the analysis on the protein crosstalk network and was constructed with PPI database. Each knot in the PPI network constructed with 181 DEGs was evaluated with the centiscape of cytoscape for the topo-characteristics, namely, Degree, Eigenvector Centrality, and Betweennesss.

Degree was the most direct and classical index evaluating the regulatory and importance of knot, which was defined as the nodes directly connected to a given node.

Eccentricity $\;C_{ecc}\left(v\right)$ represent the reciprocal inverse of the longest path between the knot v and all other knots. The eccentricity of a node in a biological network can be interpreted as easiness of a protein to be functionally influenced by all other proteins in the same network.

$C_{ecc}\left(v\right)=\frac{1}{max\left\{dist\left(v,\;w\right)\;:\;w\;\in \;V\;\right\}}$ , in which v and w were the nodes in network (V)

S.-P. Betweenness ${\text{ C}}_{\text{spb}}\left(\text{v}\right)$ represents the ratio of the path number connecting the knot s and t through v to the total number of path. A high S.-P. Betweenness score meant that the node, for certain paths, was crucial to maintain node connections.

$C_{spb}\left(v\right)=\underset{s\neq v\in V}{\sum }\underset{t\neq v\in V}{\sum }\frac{\sigma_{st}\left(v\right)}{\sigma_{st}}$ , in which s, t and v were nodes in network (V)

Our purpose was to find out the knot with the higher values on the topo-characteristics, because the higher the value the more significant it was. Since the relative significance of Degree was higher than Eigenvector Centrality, and the Eigenvector Centrality equaled Betweenness, we selected the first 40 knots with the higher Degree. Then, we selected the first 20 DEGs with the higher Eigenvector centrality and Betweenness, respectively. The thresholds of 40 and 20 were set empirically, and had no effect on the outcomes, because the key knots with the higher values of the topo-characteristics would vary with the threshold. According to the descending order of Degree, the first 40 candidate genes were selected. According to the descending sequence of Eigenvector Centrality and Betweenness, the first 20 genes were selected from the 40 candidates (Supplementary Table S6). Finally, 12 genes included in both above populations were set up as the hub genes. KEGG database was applied for pathway correlation, CluoGO for visulization, and all the manipulations were based on Cytoscape. From the DEGs by comparing the A3 to A1 subtype of the two HNSCC cohorts, 41 genes were selected for the protein crosstalk network constructed with PPI database.

### Pathway Enrichment Analysis

The DEGs from the comparison between the A3 and B subtype in both the TCGA HNSCC and GEO65858 cohorts were applied for GSEA enrichment. Then, the Enrichment map was visualized and annotated. Sample Gene Set Enrichment analysis (ssGSEA) was performed on the TCGA HNSCC, GSE65858, GSE39366, GSE117973, GSE40774, and GSE41613 cohorts with “GSVA” R package to grade the 29 immune signatures (He et al., 2018).

$$\text{Fold}-\text{Change}=\frac{1}{\text{n}1}\underset{\text{i}\in \text{immune hot}}{\sum }{\text{immune related score}}_{\text{i}}-\;\frac{1}{\text{n}2}\underset{\text{i}\in \text{immune cold}}{\sum }{\text{immune related score}}_{\text{i}}$$

Where n1 and n2 were the number of immune hot and immune cold samples, respectively. Immune related score was the sum of 22 immune cells scores obtained by Cibersort and 29 immune signature scores obtained by GSVA.

### The GSVA Scores of the Four Chemokines and the Confirmation of the Immune Hot and Immune Cold Subtype

According to the mRNA levels of the four chemokines, CXCL9, CXCL10, CXCL11, and CCL5, TCGA HNSCC, GSE65858, GSE39366, GSE117973, GSE40774, and GSE41613 cohorts were applied for ssGSEA with GSVA in R package and divided by the median grade into the high- and low-graded subtype, namely, the immune hot and immune cold subtype.

### Survival Analysis

The survival curve was generated by “Survminer” R package. The statistical differences among the immune subtype of TCGA HNSCC and GSE65858 cohorts were obtained by log rank test.

### Construction of Neural Network

By “neurnet” R package, a neural network containing an input layer, two hiding layers (there were 20 neurons in the first layer, and five neurons in the second layer. Both layers were in dropout), and an output layer.

### Activation Function: 

$\frac{1}{1+e^{-x}}$ ; Loss Function: $\frac{1}{N}\underset{i}{\sum }-\left(y_{i}*\log\left(p_{i}\right)+\left(1-y_{i}\right)*\log\left(1-p_{i}\right)\right)$

#### Statistical Analysis

All the statistical analyses were performed with R software version 4.0.4. The *t* test and Wilcoxon test were applied for the comparison between two subtypes, while ANOVA was used for comparison among more than two subtype. Fisher exact test was applied for the classified variations between and among subtypes. Pearson or Spearman coefficients were applied for the relevance between two variations. All statistical tests were two-sided and when *p* <0.05, the difference was regarded as significant.

## Results

### The Heterogeneity of Tumor-Associated Monocyte/Macrophage in Squamous Cell Carcinoma of Head and Neck

**FIGURE 1:**
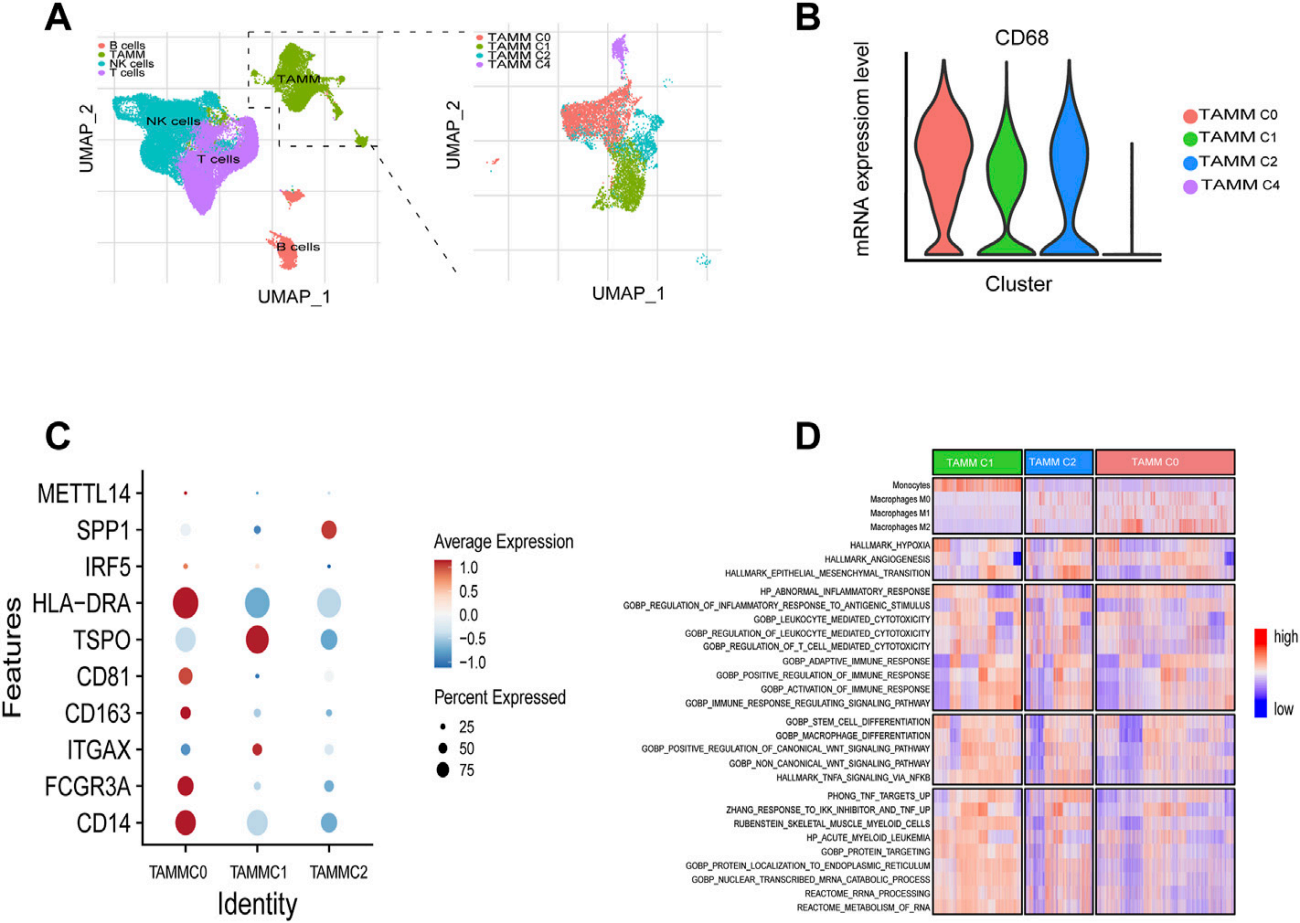
Cellular heterogeneity of tumor-associated monocyte/macrophages/macrophages at the single cell level. **(A)** Umap plots of all clusters annotated by SingleR. **(B)** CD68 expressions levels of TAMM sub-clusters (C0, C1, C2, C4). **(C)** CD14, CD16, ITGAX, CD201, CD81, TSPO, HLA-DRA, IRF5, SPP1, and METT14 expressions levels of TAMM sub-clusters (C0, C1, C2). **(D)** GSVA revealed the enrichment scores of TAMM sub-clusters in the pathways of tumor invasion, immunity, stemness, and metabolism.

A schematic diagram of the study design and principal findings is shown in Supplementary Figure S1. To classify the TAMM in HNSCC according to their differentiation status, 19,718 genes were selected from 39,711 leukocytes of HNSCC patients (Supplementary Figures S2A, S2B) qualified for dimensionality reduction with PCA and UMAP (Uniform Manifold Approximation and Projection for Dimension Reduction). The cluster classification analysis with K-NN gave rise to four clusters, which were annotated as NK cells (n = 14,925), T cells (n = 14,073), B cells (n = 2,789), and tumor-associated monocyte/macrophages cells (TAMM; n = 7,924) by SingleR. The 7924 TAMM were further classified with K-NN into seven clusters, in which the cluster 0, 1, 2, and 4 were annotated by SingleR as tumor-associated monocyte/macrophages cells (n = 6,875; Figure 1A), while the cluster 3 (n = 312), 5 (n = 478), and 6 (n = 259) as T cells and B cells (data not shown). In the TAMM clusters, TAMMC0, TAMMC1, and TAMMC2 were regarded as TAMM because of the higher CD68 expression, while the TAMMC4 with the lower CD68 expression was considered as dendritic cells (Figure 1B). Furthermore, the mature TAM-related genes, such as CD206, CD81 (marker of macrophage M2), TSPO, HLA-DRA, IRF (marker of macrophage M1), and METTL14 (C1q+), were mainly expressed in TAMMC0 and TAMMC2 (the expression in TAMMC0 was higher than that in TAMMC2), but almost silenced in TAMMC1 (Figure 1C), indicating TAMMC0 as the mature TAM, TAMMC1 as the early tumor-associated monocytes (TAM-M0), and TAMMC2 as the transforming TAM-M0 from monocytes to macrophages. Differential gene expression analysis between TAMMC1 and TAMMC0 classified 54 genes highly activated in TAMMC1, including S100A12, S100A8, VCAN, PTGS2, and CD55, into the early group of TAMM differentiation, and the other 51 genes robustly expressed in TAMMC0, such as C1QB, C1QC, MMP12, and SPP1, into the late group. Such a difference was also proven by pseudotime clustering heat map (Figure 1D, Supplementary Figure S1C). By analyzing the scRNA-seq data with GSVA and CIBERSORT, the gene function in TAMMC1 was enriched in cellular toxicity and immune inflammation, as well as the stemness and metabolism. In contrast, the gene function in TAMMC0 was less enriched in stemness and metabolism, but more highly enriched in cellular toxicity and immune inflammation, as well as the pathways of hypoxia and angiogenesis. The enriched gene function of TAMMC2 was medially located between TAMMC0 and TAMMC1 (Figure 1D; Supplementary Figure S3). Therefore, TAMMC1 was highly scored as early tumor-associated monocyte/macrophages and TAMMC0 as mature macrophages.

### Differentiation Trajectory and Copy Number Variation Verified the Heterogeneity of Tumor-Associated Monocyte/Macrophage

**FIGURE 2:**
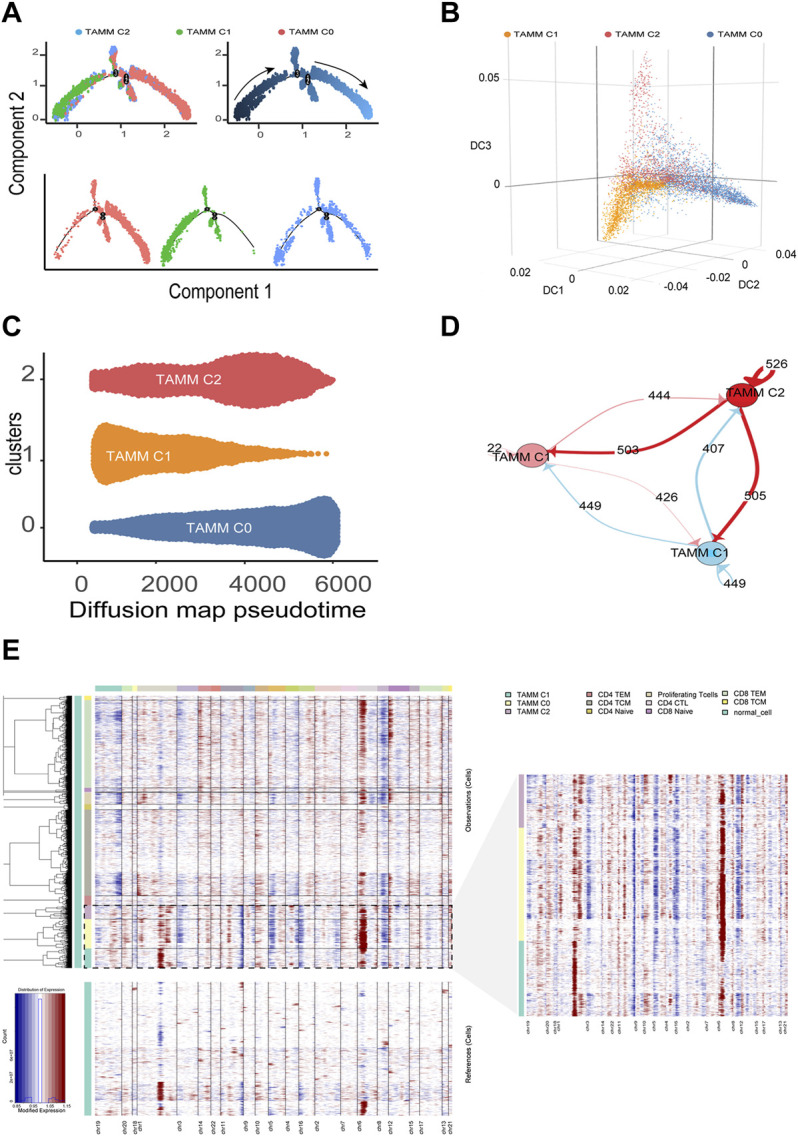
Differentiation trajectory and CNV changes of TAMM. **(A)** Monocle2 reveals the differentiation trajectory of TAMM. **(B)** Three-dimensional diffusion map embedding of macrophages reveals the different differentiation states of TAMM sub-clusters. **(C)** Density diffusion maps model revealed the content of sub-clusters of TAMM at pseudo-time. **(D)** Cell-Cell interaction network of different TAMM sub-clusters, node represent TAMM sub-cluster, and the number of lines represent ligand interactions between two sub-clusters. **(E)** Heatmap of the inferred CNV in which genes were sorted by genomic location.

According to the above differential gene expression, the differentiation trajectory of TAMM was established, in which TAMMC1 was located in the early stage, TAMMC0 in the late stage, and TAMMC2 diffusely distributed in the early and late stages (Figure 2A). Along with the time progression, TAMMC1 was decreased with the increase of TAMMC0, while TAMMC2 was increased and then decreased in the diffusion maps (Figures 2B,C). In the cell communication network, the centrally located TAMMC2 exhibited a strong connection with both TAMMC0 and TAMMC1 (Figure 2D), which coincided with the finding that TAMMC2 was located medially between TAMMC0 and TAMMC1 in the differentiation trajectory. Similarly, CNV assay revealed that the copy number and deficiency in TAMMC1 genome were relatively lower compared to those in TAMMC0 and TAMMC2 (Figure 2E). Therefore, in the heterogeneous TAMM subpopulations of HNSCC, both the gene expression profile and genomic properties indicated that TAMMC0 represented the mature TAM, TAMMC1 stood for the early differentiating monocytes, and TAMMC2 was the monocytes transforming into macrophages.

### A Criterion Classifying Squamous Cell Carcinoma of Head and Neck With Different Immune Phenotypes and Prognosis by Combining Tumor-Associated Monocyte/Macrophage Differentiation and Immune cells

**FIGURE 3:**
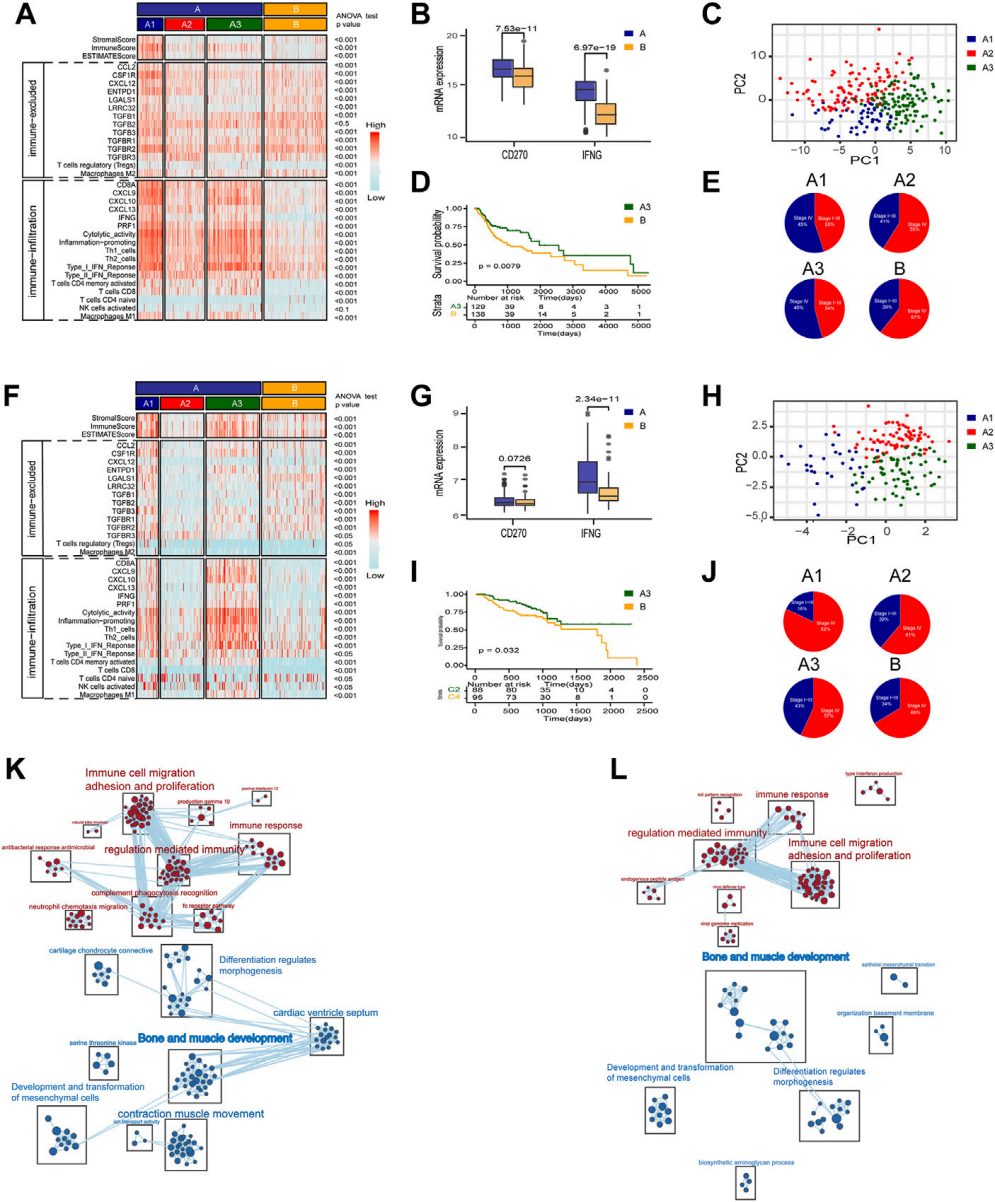
A two-step molecular classification combining the early differentiation features of TAMM and 22 immune cell scores. **(A,F)** Heatmaps of immune-related components, stromal score, and tumor purity score of the HNSCC subtypes in TCGA HNSCC **(A)** and GSE65858 cohorts **(F)**. **(B,G)** IFNG, PD-L1 expression level of subtype of TCGA HNSCC **(B)** and GSE65858 **(G)**. **(C,H)** PCA of the mRNA expression of 54 early differentiation feature genes from the HNSCC patients in the TCGA **(C)** and GSE65858 cohorts **(H)**. **(D,I)** Kaplan-Meier curves for overall survival (OS) of all HNSCC patients in TCGA **(D)** and GSE65858 **(I)** within A3 and B subtypes. **(E,J)** The pie chart showed the proportion of TMN stages with four different immunophenotypes in TCGA **(E)** and GSE65858 cohorts **(J)**. **(K,L)** GSEA network of DEGs in A3 vs B subtypes using Enrichment map in TCGA **(K)** and GSE65858 cohorts **(L)**.

As mentioned above, to explore the heterogeneity of TAMM in HNSCC patients, we identified 54 genes as the signature of early TAMM differentiation, and another 51 genes as the signature of late TAMM differentiation. To disclose the correlation of TAMM differentiation with the immune phenotypes of HNSCC, we combined the differentiation signatures with 22 immune cells to form a two-step classifier. First, the scores of 22 immune cells estimated by CIBERSORT were applied for the unsupervised clustering. Both the TCGA HNSCC and the GSE65858 cohorts were classified into A and B subtypes (TCGA HNSCC A = 265, B = 138; GSE56858 A = 175, B = 95) (Supplementary Figures S4A, S4D). The PD-1L and IFNG expression was higher in the A subtype than those in B subtype in both cohorts (*p* < 0.001, GSE65858: PD-1L *p* < 0.1). Second, unsupervised clustering was performed in the A subtype with the 54 genes as the early TAMM differentiation signatures and the 51 genes as the late TAMM differentiation signatures. The unsupervised clustering with the early TAMM differentiation signatures could divide A subtype into three subtypes (TCGA HNSCC A1 = 40, A2 = 96, A3 = 127; GSE65858 A1 = 32, A2 = 74, A3 = 69) with different clinical outcomes and immune signatures (Supplementary Figures S4B, S4C, S4E, S4F). Interestingly, the three subtypes from A subtype also showed the distinct immune infiltration and immune excluded signatures. Both the immune and stromal scores of the A1 and A3 subtypes were significantly increased compared to those in A2 and B subtypes (Supplementary Figure S5A). Moreover, the A1 subtype exhibited the stronger immune infiltration and immune excluded signatures, the A2 subtypes in both cohorts displayed the weaker immune infiltration and immune excluded signatures, while both the A3 subtypes possessed the stronger immune infiltration signatures and the weaker immune excluded signatures. In contrast to A subtype, the B subtype were weaker in immune infiltration signatures and stronger in immune excluded signatures (Figures 3A,B,F,G). The PCA with the 54 early TAMM differentiation signatures also supported this notion (Figures 3C,H). On the other hand, the unsupervised clustering with the late TAMM differentiation signatures failed to distinguish the immune phenotypes of HNSCC (data not shown). Thus, the A3 subtypes were defined as the high immune infiltration type, and the B subtype as the high immune evasion type. The following survival assay revealed the different prognoses among the subtypes, especially between A3 and B subtype (*p* < 0.05; Figures 3D,I). According to TMN staging, the A3 subtypes of both cohorts exhibited a lower ratio of IV stage patients compared to other subtypes (Figures 3E,J). To further verify the correlation between TAMM differentiation and immune phenotypes, we performed GSEA analysis on the differentially expressed genes between the A3 subtype and B subtype. The genes highly expressed in the A3 subtype were enriched in immune-associated pathways, such as activation of immune cells, adherence, proliferation, immune response, and regulation, while the genes highly expressed in the B subtype were enriched in cellular development and ECM-related pathways, for instance, mesenchymal development, pattern formation, and cytodifferentiation (Figures 3K,L). These results suggested that the early differentiation signatures of TAMM were associated with the HNSCC immune phenotypes and prognosis.

### Multiomic Characteristics Associated With the Immune Phenotypes of the Different Squamous Cell Carcinoma of Head and Neck Subtypes

Finally, CNV, SNP, and methylation levels were examined to further explore the immune phenotype in the subtype of TCGA HNSCC cohort. It was found that the mutation frequency of tumor mutation loading and tumor driver genes (TP53, TTN, etc.) in the A2 subtype was noticeably higher than that in other subtypes (Supplementary Figures S5B, S5C). CNV analysis found that the focal copy numbers in 3p, 11q, and 2p were significantly distinguishable between the A3 subtype and B subtype (Supplementary Figures S5D–E). The methylation assay revealed that there were 96 genes highly expressed in B subtype overlapped with the methylation probe highly expressed in A3 subtype, while only 13 genes highly expressed in A3 subtype were detected by the methylation probes highly expressed in B subtype (Supplementary Figures S5F–G). These findings implicated that CNV, SNP, and methylation levels also contributed to the different immune phenotypes in the HNSCC subtypes.

### Construction of Gene Regulatory Network Based on Differential Genes Between Squamous Cell Carcinoma of Head and Neck Subtype

**FIGURE 4:**
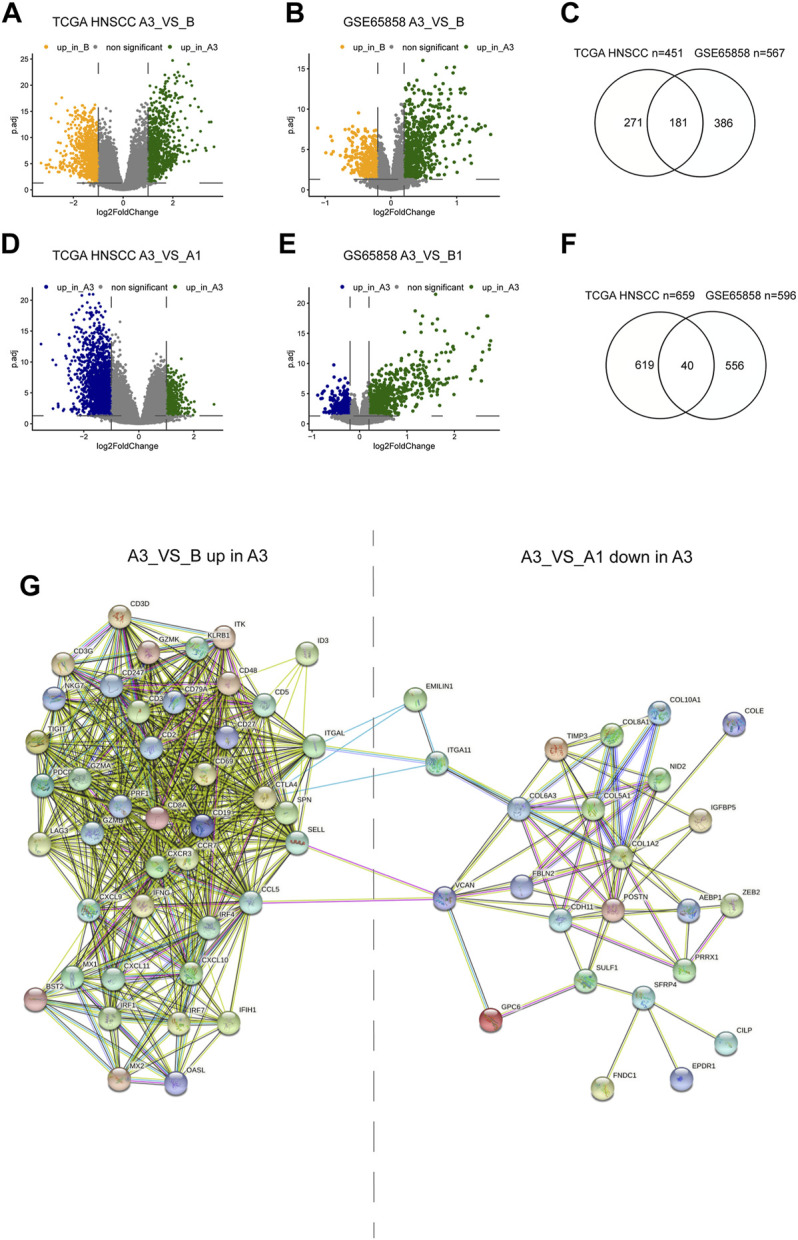
Differential genes between subtypes and protein regulatory network. **(A,B,C)** Volcano plot and Venn diagram show DEGs of A3 vs B in TCGA HNSCC and GSE65858 cohorts. **(D,E,G)** Volcano plot and Venn diagram showed the DEGs of A3 vs A1 in TCGA HNSCC and GSE65858 cohorts. **(F)** PPI protein regulatory network of 181 overlapping DEGs up in A3 (A3 vs **(B)** and the 40 overlapping DEGs down in A3 (A3 vs A1).

To explore the mechanisms regulating the formation of different subtypes, we compared the differential expressed genes (DEGs) between A3 and B subtypes, and between A3 and A1 subtypes. There were 451 highly DEGs in the A3 subtype compared to the B subtype in the TCGA HNSCC cohort (Figure 4A), and 567 highly DEGs in the A3 subtype compared to the B subtype in GSE65858 cohort (Figure 4B). There were 181 overlapped genes in the two groups of the highly DEGs (Figure 4C), which represented the high immune infiltration associated genes in HNSCC. On the other hand, we obtained 659 lowly DEGs in the A3 subtype from the comparison to the A1 subtype of TCGA HNSCC cohort (Figure 4D), and 596 lowly DEGs in the A3 subtype from the comparison to the A1 subtype of GSE65858 cohort (Figure 4E). In the two groups of lowly DEGs, 41 genes were overlapped (Figure 4F). Thus, the high immune infiltration-associated genes overlapped evidently between different cohorts, while the immune evasion-related genes showed diversity between different cohorts even in the instance of high immune infiltration. By exploiting STRING database, the 181 highly and 41 lowly DEGs were constructed into a protein crosstalk net (Figure 4G). Moreover, in the 41 immune evasion-related genes, those correlated with the genes encoding extracellular matrix (POSTN, COL6A3, COL1A2, etc.) resided in the core of the network.

### Tumor-Associated Monocyte/Macrophage-Associated Chemokines-CXCL9, CXCL10, and CXCL11-and Inflammatory Chemokine- CCLL5 Were Key Nodes in Gene Regulatory Network

**FIGURE 5:**
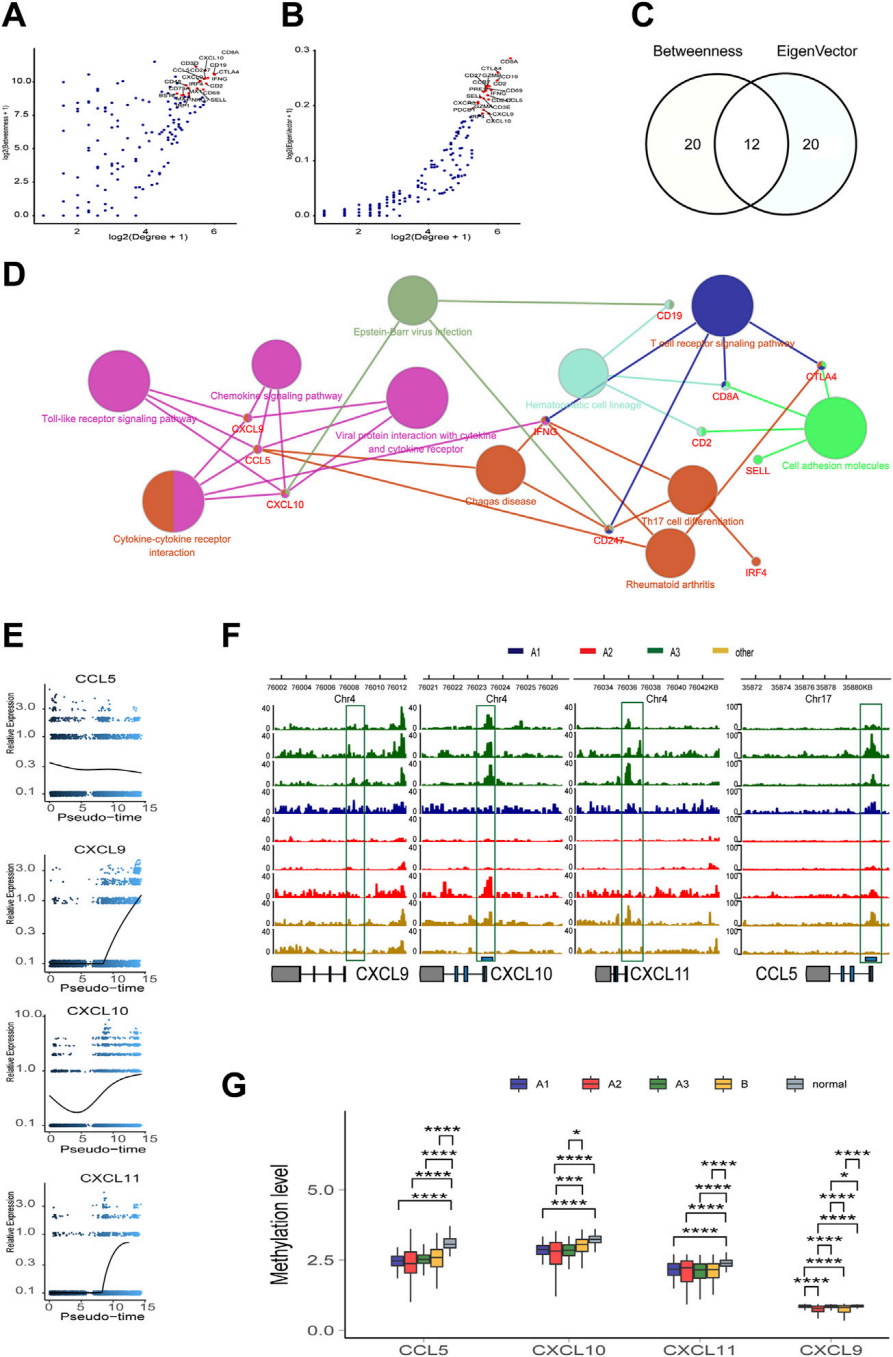
The topological feature of the network composed of 181 genes. **(A)** Dotplot of -log2(Degree+1) and log2(Betweenness+1) in selected 181 nodes. **(B)** Dotplot of log2(Degree+1) and log2(Eigenvector+1) in selected 181 nodes. **(C)** Venn diagram of top 20 genes in A or **(B) (D)** KEGG pathway association network of 12 hub genes. **(E)** Trend of mRNA expression levels of the four chemokines (CXCL9,10,11, and CCL5) following the differentiation trajectory of TAMM. **(F)** Aggregation of ATAC-seq peaks of the four chemokines of nine patients in the transcription initiation region. **(G)** Methylation levels of the four chemokines in different HNSCC subtypes.

To screen out the key nodes in the gene regulatory network of the high immune infiltration we assumed three criteria: at the center of the regulatory network, belong to the same pathway, and highly correlated expression. In the network constituted by the 181 highly DEGs, we screened out 40 highly regulated genes with the Degree more than 30. Although the correlation matrix also verified the high association among the 40 highly regulated genes (Supplementary Figure S6A), the Degree and correlation are insufficient for the identity of the key genes. Thus, the 40 candidate genes were arranged in the order of Betweenness which represented the center value of the node (Figure 5A) and Eigenvector according to the importance of integrating adjacent nodes (Figure 5B), respectively. Then, by comparing the first 20 genes arranged with Betweenness to the first 20 genes arranged with Eigenvector, 12 overlapped genes were chosen as the hub genes (Figure 5C). Finally, the function of these 12 hub genes were applied for KEGG pathway correlation with CluoGO (Figure 5D) through which four chemokines (CCL5, CXCL9, CXCL10, and CXCL11) were screened out. Although not included in the 12 hub genes, CXCL11 shared the same family with CXCL9 and CXCL10, and was highly correlated with their expression levels. So CXCL11 was also identified as one of the driver genes. These four chemokines were regarded as the key node in gene regulatory network of the high immune infiltration, because of these characteristics: 1) the core genes with the higher Degree, Betweenness, and Eigenvector in the network; 2) robust relevance at the transcription level (Supplementary Figures S6B–D), and the remarkably higher difference in CXCL9, CXCL10, and CXCL11 (A3 vs B) than the other eight genes (Supplementary Figure S6E); and 3) belong to the same pathway and share the higher topological signs. Moreover, during TAMM differentiation, CXCL9, CXCL10, and CXCL11 were increased with the time progression in Pseudotime analysis (Figure 5E). Taken together, the four chemokines with the strongest functional co-regulation and co-expression could be regarded as the pivotal genes screening the high immune evasion of HNSCC.

### The Transcription of the four Chemokines-CXCL9, CXCL10, CXCL11, and CCLL5-Was Influenced by Epigenetic and Health Factors

To further explore the endogenous (epigenetic) and exogenous (health manner) factors impacting the expression of the four chemokines-CXCL9, CXCL10, CXCL11, and CCL5--the chromatin accessibility and the methylation status at the transcription initiation regions of CXCL9, CXCL10, CXCL11, and CCL5 were analyzed in the whole genome with TCGA HNSCC ATAC and methylation database. The enriched reads were evidently concentrated at the transcription initiation regions of CXCL9, CXCL10, CXCL11, and CCL5 in the A3 subtype, compared to the A1 and A2 subtypes (Figure 5F), implicating a more active transcription of CXCL9, CXCL10, CXCL11, and CCL5 in the A3 subtype. In contrast, the methylation levels of CXCL9, CXCL10, CXCL11, and CCL5 showed insignificant difference among the subtype, implying that the four chemokines were epigenetically regulated by the manners rather than DNA methylation. However, the methylation of CXCL9, CXCL10, CXCL11, and CCL5 in the control patients were higher (Figure 5G), suggesting de-methylation of the four chemokines was crucial for HNSCC genesis. We also found that the transcription and methylation levels of CXCL9, CXCL10, CXCL11, and CCL5 were correlated with age, smoking, alcohol consumption, and HPV infection. The higher mRNA levels of the four chemokines were detected in the HNSCC population with older age and lower consumption of tobacco and alcohol (Supplementary Figure S7A, S7C, S7E; *t* test, *p* < 0.05). The HPV positive HNSCC group exhibited a higher CXCL10 mRNA level and an increased methylation of CXCL9 and CXCL11 compared with the HPV negative HNSCC group (Supplementary Figures S7B, S7D; *t* test, *p* < 0.05). Thus, it was concluded that, although not associated with the immune phenotypes of HNSCC, the transcription of the four chemokines regulated by DNA methylation and health factors were critical for HNSCC genesis.

### The Four Chemokines-CXCL9, CXCL10, CXCL11, and CCL5-Positively Regulated Immune Responses and Were Associated With the Low CNV and CASP8 Mutations in the Squamous Cell Carcinoma of Head and Neck Genome

**FIGURE 6:**
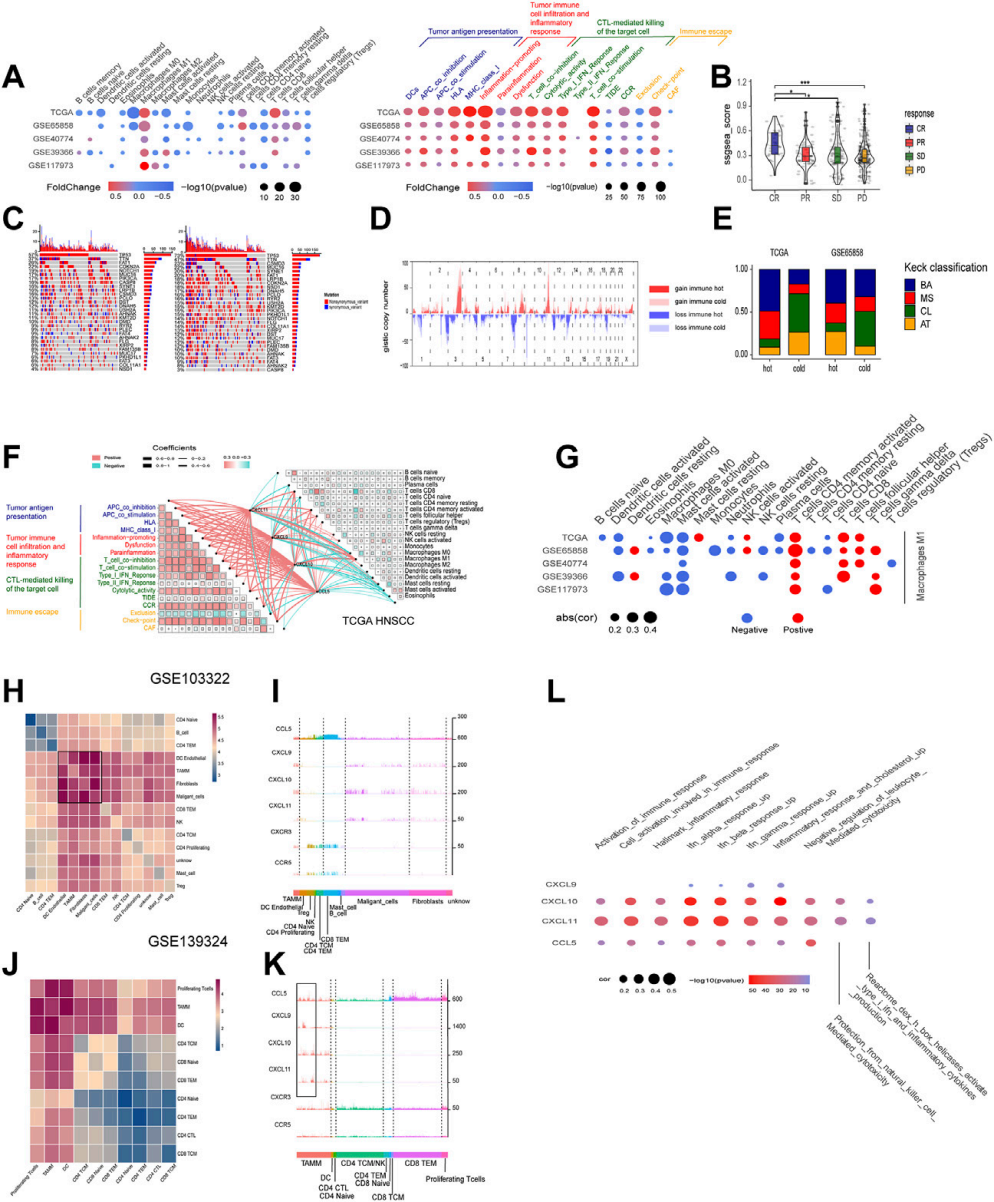
The roles of the four chemokines (CXCL9,10,11, and CCL5) in HNSCC TME and their association with tumor genomic changes. **(A)** Dotplot summarized the scores of 22 immune cells estimated by GIBESORTRT and GSVA scoring on the fold change and *p*.adjust of 29 immune signature between the immune hot and cold group classified by the median GSVA of the four chemokines (only *p* < 0.05 was given). **(B)** Vilionplot summarized the GSVA scores of CXCL9, CXCL10, CXCL11, and CCL5 of four outcomes (CR, PR, PD, SD) in the cohort receiving anti-PD-1/PD-L1 immunotherapy. **(C)** The OncoPrint was constructed between high and low scores of the top 30 genes with the highest mutation frequency. **(D)** CNV plot showed the frequency of copy-number gains (red) and deletions (blue) among immune hot and cold groups of the TCGA-HNSC cohort. **(E)** The stacking histogram showed the distribution of Keck classification in the immune hot and cold groups of TCGA HNSCC and gse65858 cohorts. **(F)** Correlation between the four chemokines and GIBESORTRT score of 22 immune cells in HNSCC-TCGA and 29 immune signature GSVA score. The cell charts in the upper-right triangular exhibited the correlation among the 22 immune cell scores, and the cell charts in the -lower triangular showed the correlation among the 17 immune signature scores (Red stood for positive and green for negative, the darkness and lightness of the colors for the high and low of the coefficients, and the size of the cell for *p* value). The lines between two cell charts represent the correlation of the mRNA of the four chemokines with the bilateral immune scores (Red stood for positive and green for negative, and the thickness of the lines for the high and low of the coefficients). **(G)** Dotplot summarized the correlation coefficients between the scores of 21 immune cells estimated by GIBESORTRT and the scores of Macrophage M1 estimated by GIBESORTRT (only *p* < 0.05 was given). **(H,J)** Heatmap of average expression level of ligand–receptor interactions in all clusters in GSE103322 and GSE139324. **(I,K)** Histogram of expression levels of CCL5, CXCL9, CXCL10, CXCL11, CXCR3, and CCR5 in each cell in GSE103322 and GSE139324 cohorts. **(L)** Dotplot summarized the correlation coefficients between the expression level of CXCL9, CXCL10, CXCL11, and CCL5, and the GSVA of 10 immune signatures in each cells classified as TAMM.

According to the mRNA levels of the four chemokines, six HNSCC cohorts (one TCGA cohort and five GEO cohorts) were graded with GSVA, and then divided into the high- and low-graded groups with the median grade to evaluate the correlation of the four chemokines with immune response. There was a remarkable difference between the high- and low-graded groups in the immune signature and genome. The TCGA and most high-graded cohorts showed a higher enrichment of immune cells (Macrophages M1, CD4 T cells memory activated, and CD8 T cell) in the infiltration grading of the 22 kinds of the immune cells, got higher scores in the enrichment of antigen present during tumor immune circle, immune cell infiltration, and the recognizing and killing of tumor cells by effector T cells in the 29 immune signatures assay (Figure 6A), and was given the lower scores in the TIDE assay. All of the results suggested a better response to immune therapy and was verified by the cohorts of immune therapy, in which the GSVA grades of the four chemokines in the CR group were higher than those in PR, SD, and PD groups (Figure 6B). Based on these findings, we classified HNSCC into the immune hot and immune cold phenotype according to the GSVA grades of the four chemokines. Then, we estimated the distribution of the mutations from the first 30 HNSCC driver genes with the highest frequency of mutation (TP53, TTN, CSMD3, SYNE1, etc.,) in the hot and cold immune groups, and found that except for CASP8, all other driver genes had an elevated frequency of mutation in the low-graded group (Figure 6C; Supplementary Table S7), implying that the mutations of CASP8 endowed HNSCC with a stronger immunity. Moreover, CNV analysis revealed that in the immune cold group, an active CNV was detected in several hot spot regions (gain: 3p, 8q, 17q, 18p. loss: 2q, 7q, 13q) (Figure 6D). In combination with Kech classification, we found that the most immune hot was BA type, while the most immune cold was CL type (Figure 6E). All the above results suggested that the four chemokines could not only act as the markers identifying the HNSCC with high concentration of immune cells (CD8 T cell, Macrophage M1, etc.), but also reflect the HNSCC characteristics comprehensively. It was also suggested that the immune hot subtype of HNSCC could enhance the immune responses through CASP8 mutations, and the immune cold subtype also circumvented immune responses through gene mutations. Further exploration on the crosstalk among the four chemokines, TME, and immune cells in the immune circle by analyzing the relevance in TCGA cohort disclosed that CXCL9, CXCL10, CXCL11, and CCL5 showed a strongly positive association with immune cells (Macrophages M1, CD4 T cells memory activate and CD8 T cells), and the three stages of tumor immune circle (Figure 6F). Since the similar association was also detected in other HNSCC cohorts, the four chemokines were proven to enhance the anti-tumor immune capability. Moreover, we also found that macrophages M1 was strongly positively associated with the activation of dormant CD8 and CD4 T cells (Figure 6G).

### The Relationship Between the Four Chemokines and the Sub-Populations in the Squamous Cell Carcinoma of Head and Neck Tumor Micro-Environment at Single Cell Level

The cohorts of GSE10332 and GSE139324 (the GSE10332 cohort contained all kinds of cells in TME, while the GSE139324 cohort only contained infiltrative leukocytes) were applied for the relationship assay at the single cell level to disclose the effects of CXCL9, CXCL10, CXCL11, and CCL5 on the HNSCC sub-populations in TME. Cell communication assay with CellPhoneDB found that TAM, DC, tumor-associated fibroblasts (CAF), and malignant epithelium cells communicated with themselves and other cells intensively (Figures 6H,J). Interestingly, CXCL9, CXCL10, and CXCL11 were expressed robustly in TAM, malignant epithelium cells, and CAFs, but weakly in CD4 and CD8 T cells. However, CXCR3, the common receptor for CXCL9, CXCL10, and CXCL11, was expressed in CD4 and CD8 T cells (Figure 6I), indicating that CD4 and CD8 T cells were chemo-attracted to immune focus by the CXCL9, CXCL10, and CXCL11 emanated from TAM, malignant epithelium cells, and CAF. Moreover, CCL5 and its receptor, CCR5, were mainly expressed in CD4, CD8 T cells, and NK cells (especially CD8 T cells; Figure 5K), suggesting that CCL5 influenced T cells through autocrine or paracrine. Worthy of note, the mRNA peaks of CXCL9, CXCL10, CXCL11, and CCL5 in TAM were distributed in multiple sub-populations (Figure 6K), suggesting that although the four chemokines were highly correlated at the bulk RNA-seq level, such correlation could be inconsistent at the single cell level due to the heterogeneity of TAMM. Finally, the relevance assay on TAM clusters at the single cell level disclosed that the mRNA levels of the four chemokines were positively correlated to the pathways involved in immune infiltration and immune inflammation response (Figure 6L). Therefore, the expression of the four chemokines were associated with the immune sub-populations in TAMM.

### The Immune Hot Subtype of Squamous Cell Carcinoma of Head and Neck Characterized by the High Expression of the Four Chemokines was Sensitive to Anti-Cancer Drug Targeting ERK1-MARK and RAS Pathway

**FIGURE 7:**
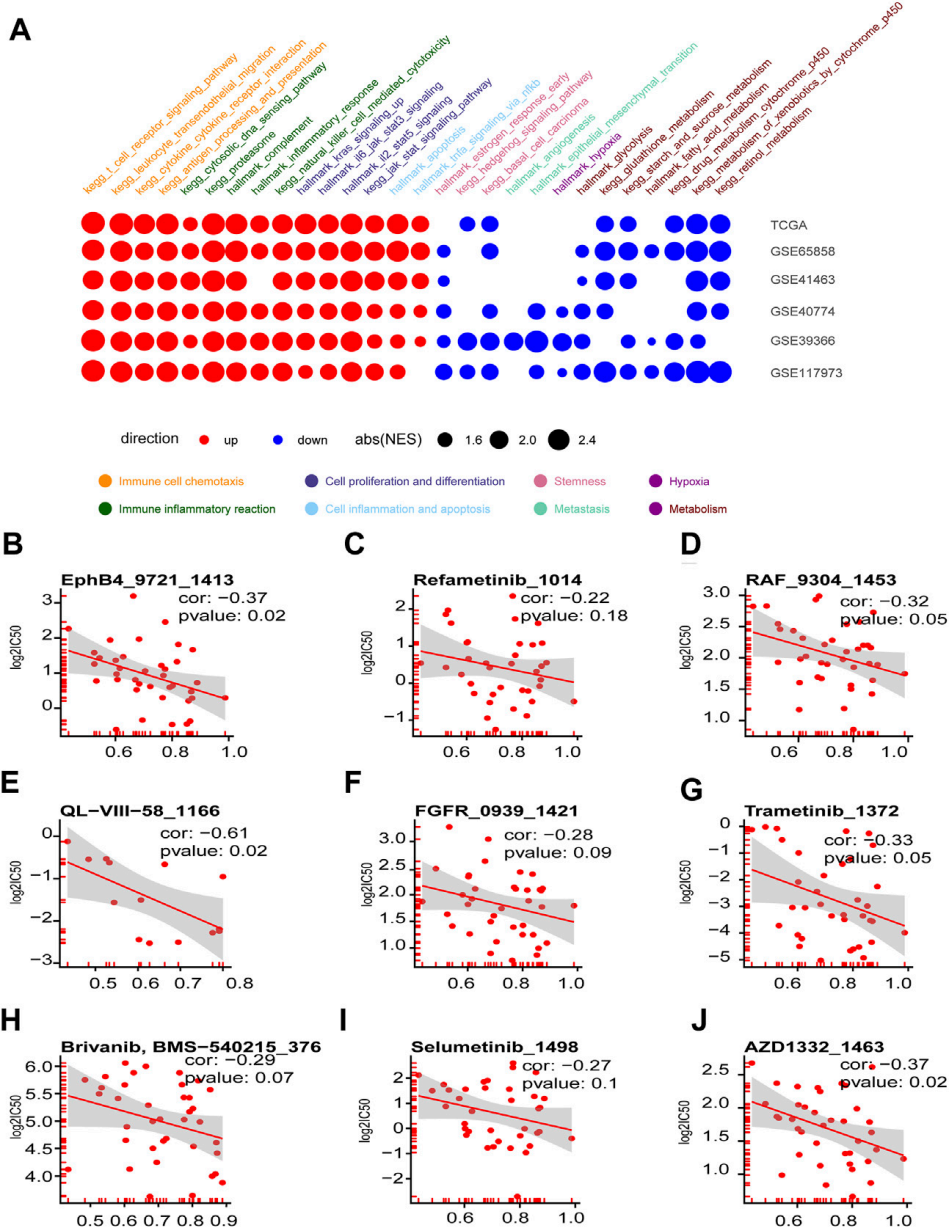
Enriched tumorigenesis pathways in immune hot and cold phenotypes determined by the four chemokines (CXCL9,10,11, and CCL5) and immune hot sensitive drugs. **(A)** Dotplot summarized the GSEA NES scores of signal pathways related to tumor immunity and tumorigenesis between immune hot and cold groups in the six HNSCC cohorts (only *p*.adjust<0.05 were given). **(B–J)** Scatter diagram summarized the correlation coefficients between GSVA score of CXCL9, CXCL10, CXCL11, and CCL5, and the log_2_(IC50) in seven drugs targeting ERK-MAPK pathway **(B–I)** and two drugs targeting RAS pathway **(I, J)**.

The GSEA assay on the DEGs between the high- and low-graded groups of the six HNSCC cohorts revealed that the pathways enriched in the high-graded groups were mainly involved in chemoattraction and immune response of immune cells, as well as cell proliferation and differentiation, which were consistent in different cohorts. In contrast, the pathways enriched in the low-graded groups were relatively sparse in different cohorts, though mainly concentrated in tumorigenesis and progression, such as stemness, metastasis, metabolism, and hypoxia (Figure 7A). The relevance assay on the IC50 of the HNSCC cell lines treated with anti-tumor drugs in GDSC database disclosed that the GSVA scores of CXCL9, CXCL10, CXCL11, and CCL5 were negatively correlated to ERK1-MARK (Figures 7B–H) and RAS pathway (Figures 7I,J) targeted by anti-HNSCC drugs, implicating that the susceptibility of the immune hot subtype of HNSCC to the drug originated from the influence on ERK1-MARK and RAS pathway.

### A Neural Network Predicting Model With the Four Chemokines and Three Immune Cells

**FIGURE 8:**
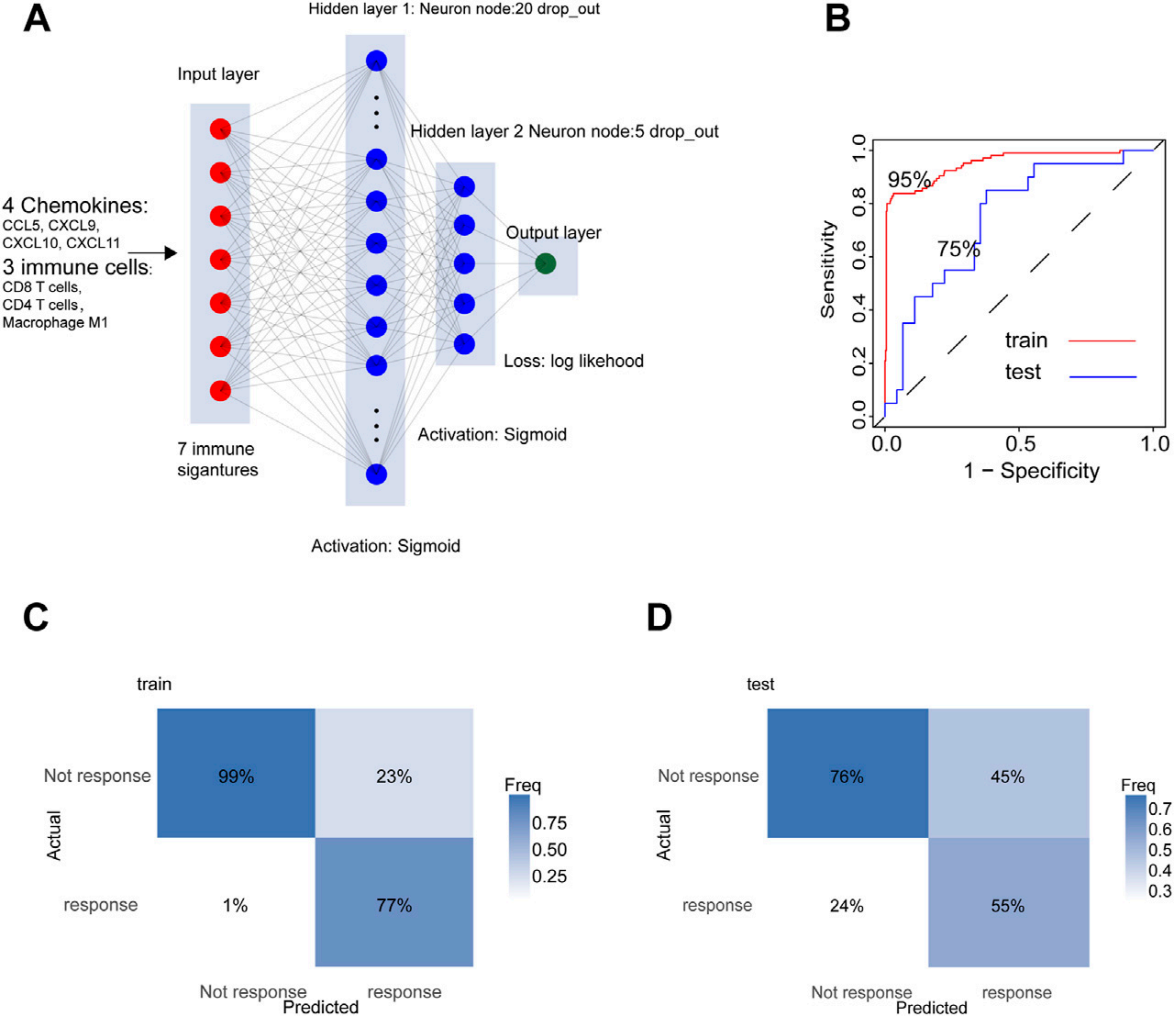
The four chemokines and three immune cells were used to construct a neural network model to predict the response to anti-PD-1/PD-L1 immunotherapy. **(A)** Schematic diagram of the neural network. **(B)** ROC plot of train cohort and test cohort. **(C,D)** The confusion matrix in test cohort and train cohort.

Through the above studies, we found out a strongly positive correlation between four chemokines (CXCL9, CXCL10, CXCL11, and CCL5) and three kinds of immune cells (Macrophage M1, CD8, and CD4 T cells) in the immune phenotypes of HNSCC. Since the above seven factors were located in the core of the immune response in HNSCC, we designed a 4-layer neural network to predict the response (response: CR, PR. not response: SD, PD) to the treatment with auti-PD-1/PD-L1 (Figure 8A), in which GSE154538, GSE141119, GSE91061, GSE78220, GSE176307, and the IMvigor210 acted as the training set (n = 501), and GSE93157 as the test set (n = 65). The AUC of the training set and the test set in the prediction model reached 95% and 74.6%, respectively (Figure 8B). The confusion matrix of the training and test sets was displayed in Figures 8C,D.

## Discussion

Despite the great progress made by ICB in multiple tumors, only a minority of HNSCC patients have benefited from ICB therapy. It is of major importance to characterize the HNSCC sub-population susceptible to ICB therapy, and the key genes maintaining the sub-population (Curran et al., 2010). Although a variety of criteria were proposed previously, few of them concern the association of TAMM with the immune responses in HNSCC. Since TAMM in TME contributed greatly to tumorigenesis and progression (Estko et al., 2015; Gao et al., 2016), a lot of researchers focused on the potentials of TAMM in immune therapy (Coifman et al., 2005). However, most previous studies and strategies ignored the heterogeneity of TAMM, but treated the TAMM as a static entity, which meant the immune therapy was challenged by TAMM heterogeneity. In the present study, we applied bioinformatical methods from multiple dimensions to explore the heterogeneity of the TAMM during differentiation, as well as its correlation with the immune responses in HNSCC. Although CIBEOSORT was not reported to be applied in the matrix of single-cell RNA-Seq, we think that, according to the resolve of non-negative matrix in the algorithm theory of CIBEOSORT, the bulk RNA-seq could be resolved into the matrix of single-cell RNA-Seq timing the matrix of cell type clusters. In our study, the single-cell RNA-Seq matrix, namely, the Leukocyte signature matrix (LM22), was set as the decision variable, and the single sample in the bulk RNA-seq as response variable for SVM linear deconvolution. The outcome weighted vector was regarded as the cell type abundance of each sample. Therefore, it is sound to transform the bulk RNA-seq matrix into Single-cell RNA-Seq matrix by treating each cell as a sample in the bulk RNA-seq. By treating the Single-cell RNA-Seq data with the above algorithm theory, we found that combining the genes characterizing early TAMM differentiation with the immune cells in HNSCC could provide a criterion classifying the immune subtypes of HNSCC more precisely. Furthermore, the four chemokines-CXCL9, CXCL10, CXCL11, and CCL5-were identified not only as the driver genes initiating and maintaining the immune hot subtype of HNSCC, but also the nodes connecting TAM (macrophage M1), CD4, and CD8 T cells together.

The heterogeneity of TAMM in HNSCC was found to result from not only the different subpopulations, but also the different stages during differentiation. Molecularly, the gene expression and genomic constitution of TAMM underwent remarkable alterations during the differentiation from tumor-associated monocyte to tumor-associated macrophages, which was also supported by the *in vitro* assay (Singhal et al., 2019). Previously, a criterion exploiting 22 kinds of immune cells was once proposed to classify HNSCC into the high and low immune enrichment subtype (Feng et al., 2020; Zhang et al., 2020), however, it was too broad to characterize the immune subtype and prognosis. To establish a practical and accurate criterion, we divided the gene expression profile during TAMM differentiation into the early and later groups, and found out the DEGs between the early and later groups. By using multiple bioinformatical methods, the DEGs in the early TAMM differentiation were found, indicating a better prognosis in the subtype with high immune infiltration and low immune evasion (Sanmamed and Chen., 2018). Thus, the DEGs in early TAMM differentiation could reflect more details in the immune infiltration, evasion (which could estimate the immune phenotype more comprehensively) (Turan et al., 2021), and prognosis of HNSCC.

Previous studies reported a correlation of the better prognosis with the elevated tumor mutation burden (TMB) (Chan et al., 2015; Spencer et al., 2016; Büttner et al., 2019). However, in our study, the A2 subtype which had the highest TMB in the TCGA HNSCC cohort displayed the lower immune infiltration and evasion, implicating the insusceptibility to ICB therapy. Since similar characteristics were also detected in another HNSCC clinical cohort (Kim et al., 2020), it was still unclear whether the HNSCC subtype with a higher TMB was susceptible to PD-1/PD-L1 therapy.

The DEGs between A3 and B subtype (high immune infiltration vs high immune evasion) were applied to construct the protein crosstalk network from which the genes highly correlated in expression at the nodes of regulatory network and sharing the same pathway were identified as the driver genes initiating and maintaining the immune subtype. The DEGs between the high immune infiltration and high immune evasion (A3 subtype vs B subtype) showed a high similarity in different cohorts. In contrast, the DEGs between high immune infiltration subtype (A1 vs A3 subtype) were different from one another in different cohorts. Therefore, we concentrated on the DEGs between the high immune infiltration and high immune evasion (A3 subtype vs B subtype) to explore the key genes regulating the immune hot phenotype. Since both the A3 and A1 subtypes stood for the high immune infiltration subtypes in the two cohorts, the difference between A3 and A1 subtypes represented the discrepancy between the high immune infiltration subtypes. From Figure 5G, it can be seen that such discrepancy could be partially attributed to the various extents of the immune evasion. However, because the number of the intersected DEGs in the A3 and A1 subtypes was too low to contribute to the difference, the factors resulting in the difference were implicated varying dramatically in different cohorts. Since the A3 and B subtypes in the two cohorts exhibited the converse immune signatures, their comparison was supposed to get the core genes associating immune infiltration with the HNSCC. The numerous overlapped DEGs from the two cohorts reflecting the similarity in the high immune infiltration between the two cohorts allowed the following exploration of the pivotal genes in the HNSCC with immune infiltration phenotype. Taking all above findings into account, we concluded that the TAMM differentiation related chemokines-CXCL9, CXCL10, and CXCL11, and inflammatory chemokine CCL5-were the driver genes initiating and maintaining the high immune infiltration phenotype of HNSCC. A series of previous reports supported that the four chemokines could work as the potential targets of immune therapy. During tumorigenesis and progression, the CXCL9-11/CXCR3 axis regulated the differentiation and chemoattraction of T cells (Tokunaga et al., 2018), and the CCL5/CCR5 axis influenced growth and metastasis (Aldinucci et al., 2020). Previous reports showed that CXCL9, CXCL10, and CCL5 could mark T cell–inflamed phenotype of pancreatic cancer (Romero et al., 2020). CXCL9 and CCL5 activated immune responses and enhanced ICB therapy in mouse model of ovary cancer (Dangaj et al., 2019), and the melanoma in CXCR3 knock-out mice exhibited decreased immune infiltration and poor prognosis (Korniejewska et al., 2011). However, we have to acknowledge that there was subjective opinion in the criteria of the pivotal genes. Actually, besides the four chemokines, the other eight candidate hub genes were also verified to act as the pivotal genes establishing and maintaining the high immune infiltration to some extent. However, we think that CXCL9, CXCL10, CXCL11, and CCL5 were the optimal combination, because they satisfied the three criteria: 1). they shared multiple pathways, implicating that they could collectively reflect the activity of immune pathway, instead of independently. Other genes were located in different pathways, which raised the uncertainty for their function, though they possessed the higher network topology; and 2). CXCL9, CXCL10, CXCL11, and CCL5 were highly correlated in expression. We tested the correlated expression of the 181 overlapped DEGs in TCGA cohorts and confirmed a higher correlation among CXCL9, CXCL10, and CXCL11 than among other DEGs. Although CXCL11 was excluded from the 12 candidate genes with the highest topological signatures, it had the higher correlated expression, belonged to the same family and shared multiple pathways with the other three chemokines. Therefore, we selected CXCL9, CXCL10, CXCL11, and CCL5 as the pivotal genes in the high immune infiltration of HNSCC for the following study.

Because the roles of the four chemokines in HNSCC have been little studied, to explore their pivotal effects in the immune responses to HNSCC, we divided the immune circle into infiltration and evasion stages. The immune infiltration included: 1) the release and convey of tumor cell antigen, 2) chemoattraction and infiltration of immune cells into TME by the cytokines and inflammation, and 3) recognition and elimination of HNSCC cells by CTL. With the exhaustion of CTL and the expression of immune suppression factors (TGF-beta, PD-L1, etc.) by HNSCC, the immune evasion commenced. The bulk RNA-seq data revealed that CXCL9, CXCL10, CXCL11, and CCL5 were positively correlated to all three steps of immune infiltration, but negatively to immune evasion. The scRNA-seq data further disclosed that the four chemokines and their receptors were highly expressed in DCs, enhancing the antigen presentation in TME; the high CCL5 expression in CD8 T cells, NK cells, and certain TAM promoted inflammation; the tumor cells highly expressing CXCL9, CXCL10, and CXCL11 attracted the CD8 T cells, NK cells, and TAM, which eliminated tumor cells by perforating cell membrane, digesting through serine proteases and apoptosis via ligand binding (Lee et al., 2018). All of these results supported the role of the four chemokines in initiating and maintaining the immune hot phenotype of HNSCC.

According to the GSVA scores of the four chemokines, the HNSCC cohorts were divided into the subtypes of immune hot and immune cold. The immune cold subtype was characterized by the evident alteration on CNV, which was correlated to tumor invasion and decreased immune responses (Davoli et al., 2017). This finding also implied that the decreased immune responses in immune cold subtype resulted from the imbalanced immune gene expression caused by CNV alteration, as opposed to the dysfunction of single immune gene. The other sign of immune cold subtype was the higher mutation frequency, including the tumor driver genes of TP53, TTN, etc. Reversely, the frequency of CASP8 in immune hot subtype was noticeably higher than that in immune cold subtype. Since several studies demonstrated that the CASP8 mutations characterized the local activation of immune cells and inflammation (Rooney et al., 2015; Tummers and Green., 2017), it suggested that CASP8 mutation was capable of activating immune responses in the immune hot subtype.

Worthy of note, the inflammation resulting from the four chemokines could also increase the tumor invasion. As shown in previous studies, the VEGF-PIK3/AKT pathway activated by CCL5 promoted tumor metastasis by stimulating angiogenesis and ECM remodeling (Karnoub et al., 2007; Wang et al., 2012), and the robust expression of CXCL10 also enhanced gastric cancer invasion and metastasis by binding the receptor CXCR3A (Yang et al., 2016). Thus, further exploration was still required to elucidate the relationship between the four chemokines and HNSCC prognosis. Additionally, the four chemokines were strongly associated with the check points on HNSCC surface, implicating that HNSCC cells could circumvent immune attacks by conveying the signal of “Don’t eat me” to CTL through the check point molecules (PD-L1, PD-1, etc.). Thus, the four chemokines might also indirectly enhance the tumor invasion and metastasis even when directly attacked by tumor cells. Since CCL5 was reported to rapidly induce cyclin D1, c-Myc, Ha-Ras through MARK-ERK, and Jak/STAT signaling, as well as glucose in-take and ATP production to stimulate tumor growth (Ding et al., 2016; D. ; Gao & Fish, 2018; Murooka, Rahbar, & Fish, 2009), the HNSCC cell lines with the high expression of the four chemokines were sensitive to the anti-tumor drug targeting MARK-ERK and RAS pathways.

The four chemokines were associated positively with Macrophages M1, activated CD4, and CD8 T cells, but negatively with Macrophages M0 (in TCGA HNSCC cohort) and tumor-associated monocyte/macrophages (GEO cohort), suggesting the lower expression of the four chemokines in the early tumor-associated monocyte/macrophages or macrophages M0, and the gradually elevated expression in differentiating TAMM. Moreover, the higher expression of the four chemokines were intensively detected in macrophage M1, instead of M2, also implicating their association with the polarization of macrophages, which was supported by the recent studies that found that the lack of CCL5 promoted the polarization in macrophages M2 (M. Li M et al., 2020), and CXCL9 and CXCL10 induced the polarization in macrophages M1 (Kohler et al., 2019). The scRNA-seq revealed that the mRNA peaks of the four chemokines in TAM were distributed in different subpopulations of TAMM, suggesting that the strong and exclusive co-regulation of the four chemokines disclosed by the bulk RNA-seq were inconsistent with the single cell level, which required further investigation.

In the immunotherapy cohort receiving anti-PD-L1/PD-1, the mRNA levels of the four chemokines were elevated significantly in CR group, verifying their positive roles in immune responses. It also encouraged us to establish a criterion predicting the patients’ responses to ICB therapy. In the premise of the substantial immune capacity of resisting tumors, ICB therapy facilitated T cells to eliminate tumor cells by blocking immune evasion, namely, the validity of ICB therapy depended on the patients’ immunity. Considering the crucial roles of the four chemokines (CXCL9, CXCL10, CXCL11, and CCL5) and the three pivotal immune cells (Macrophages M1, CD 4, and CD8 T cells) in tumor immunity, we established a criterion predicting the validity of ICB therapy through neural network, in which the AUC in training and test sets achieved 100% and 74%, respectively.

We have to acknowledge the shortcomings in this study. First, although they have been verified in other tumors, work is still required to verify the roles of the four chemokines in HNSCC found by the bioinformatical analyses. Second, the exploration on the mechanisms resulting in the immune evasion in the high immune infiltration subtype was insufficient. Third, the predicting and verifying cohorts were not HNSCC cohorts, which might weaken the prediction accuracy in HNSCC cohorts because of the variations among tumors. Fourth, despite the impressive promotion in tumor immunity, the roles of the four chemokines-CXCL9, CXCL10, CXCL11, and CCL5-in tumorigenesis and progression were still in debates.

## Conclusion

In summary, we indeed established the core roles of the four chemokines-CXCL9, CXCL10, CXCL11, and CLL5-in HNSCC immunity by combining TAMM differentiation and HNSCC TME. From the perspective of the four chemokines associating TAMM with HNSCC immunity, we found the limitation of treating TAM as a static entity and the potential values of the four chemokines in tumor immunity. Considering the few studies on HNSCC immunity, our present study provided bio-informatics support for future explorations.

## Data Availability

The datasets presented in this study can be found in online repositories. The names of the repository/repositories and accession number(s) can be found in the article/Supplementary Material.
