# Supplementary figures and images for: The Chemokines Initiating and Maintaining Immune Hot Phenotype Are Prognostic in ICB of HNSCC
Source: Front Genet. 2022 May 27;13:820065. doi: 10.3389/fgene.2022.820065 (PMC9186378; doi:10.3389/fgene.2022.820065)

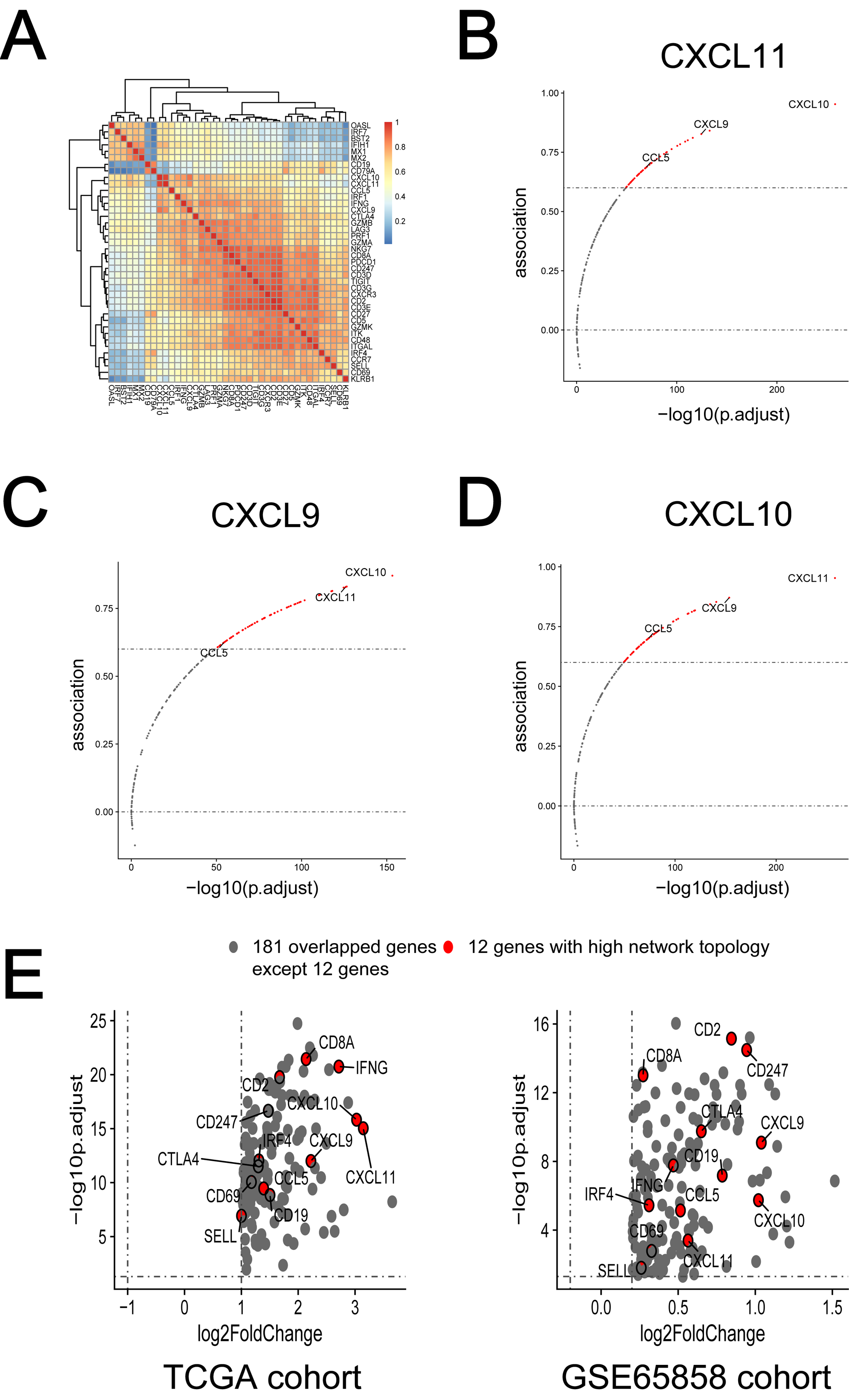

Supplement: Supplementary file 3 [file Image6.TIF]

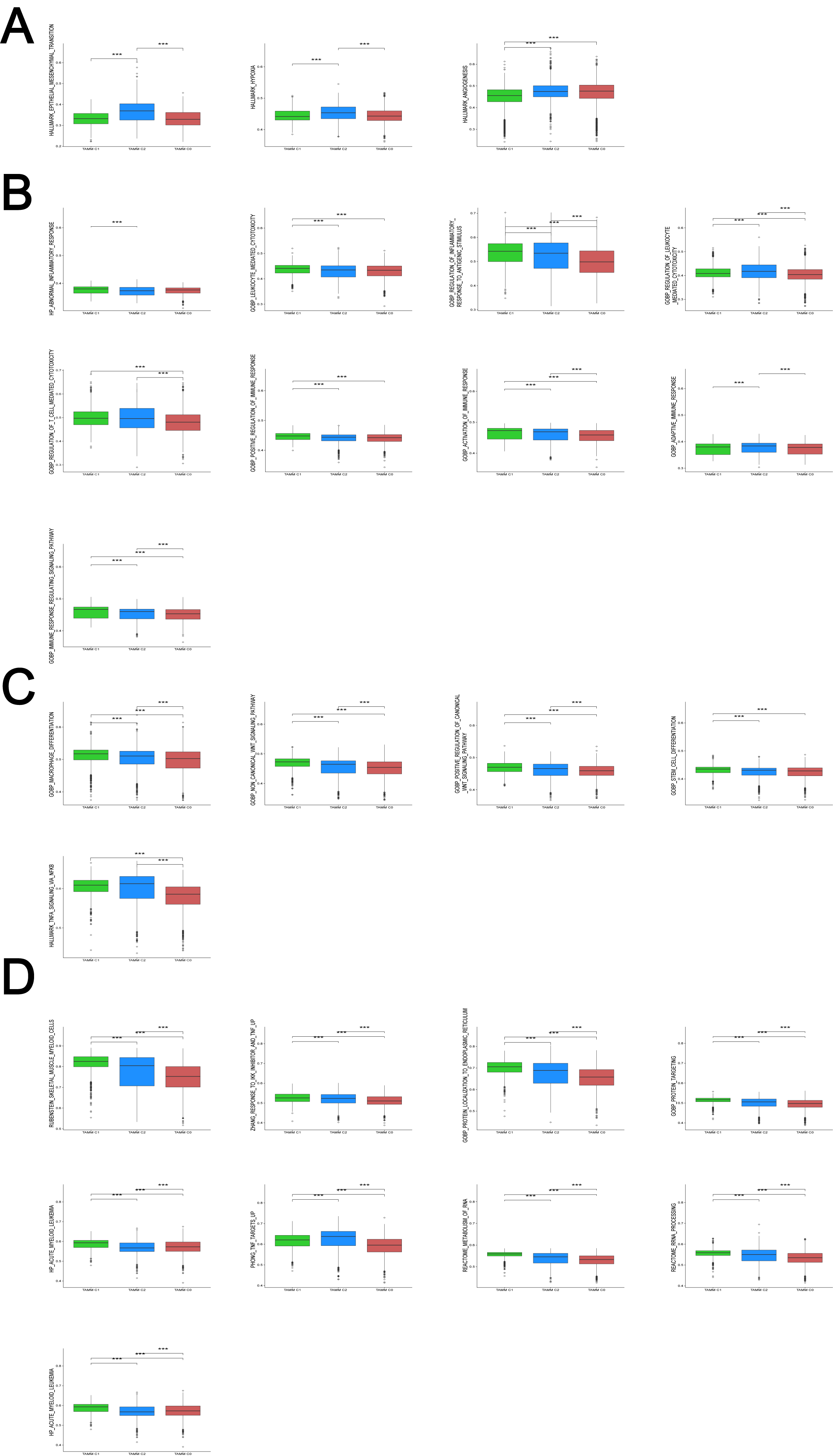

Supplement: Supplementary file 4 [file Image3.TIF]

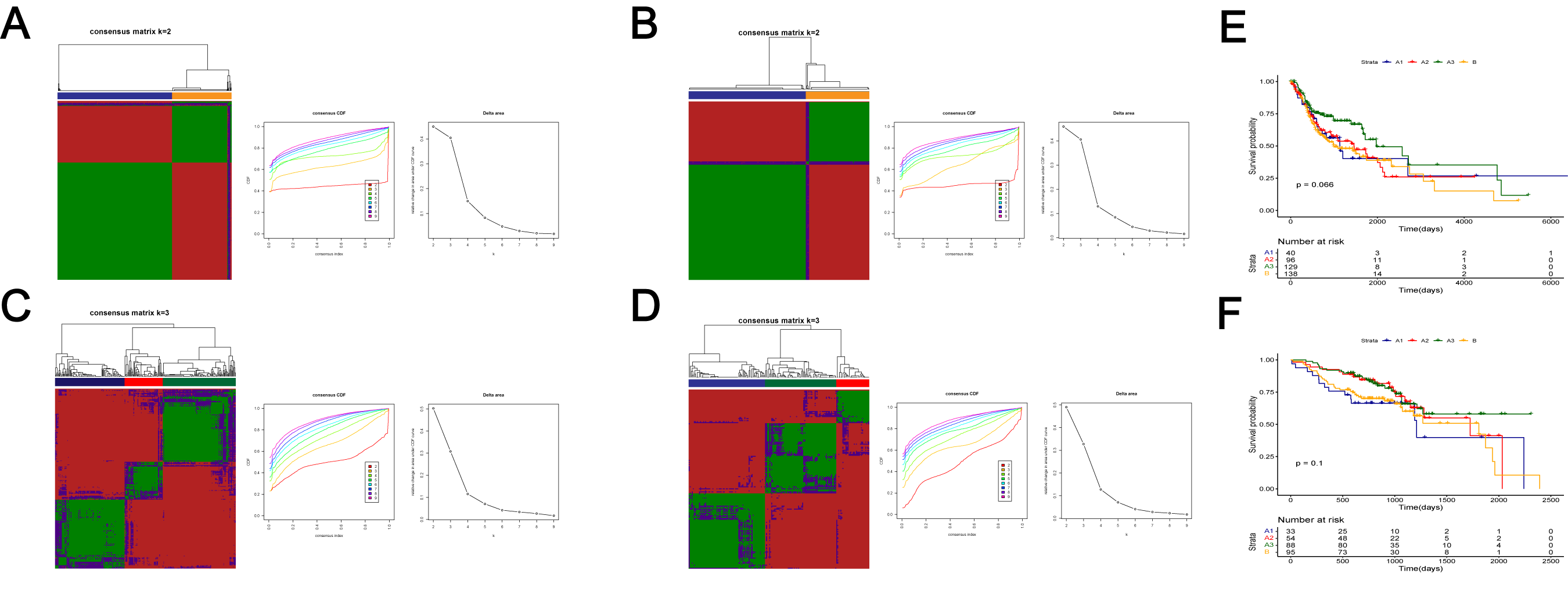

Supplement: Supplementary file 5 [file Image4.TIF]

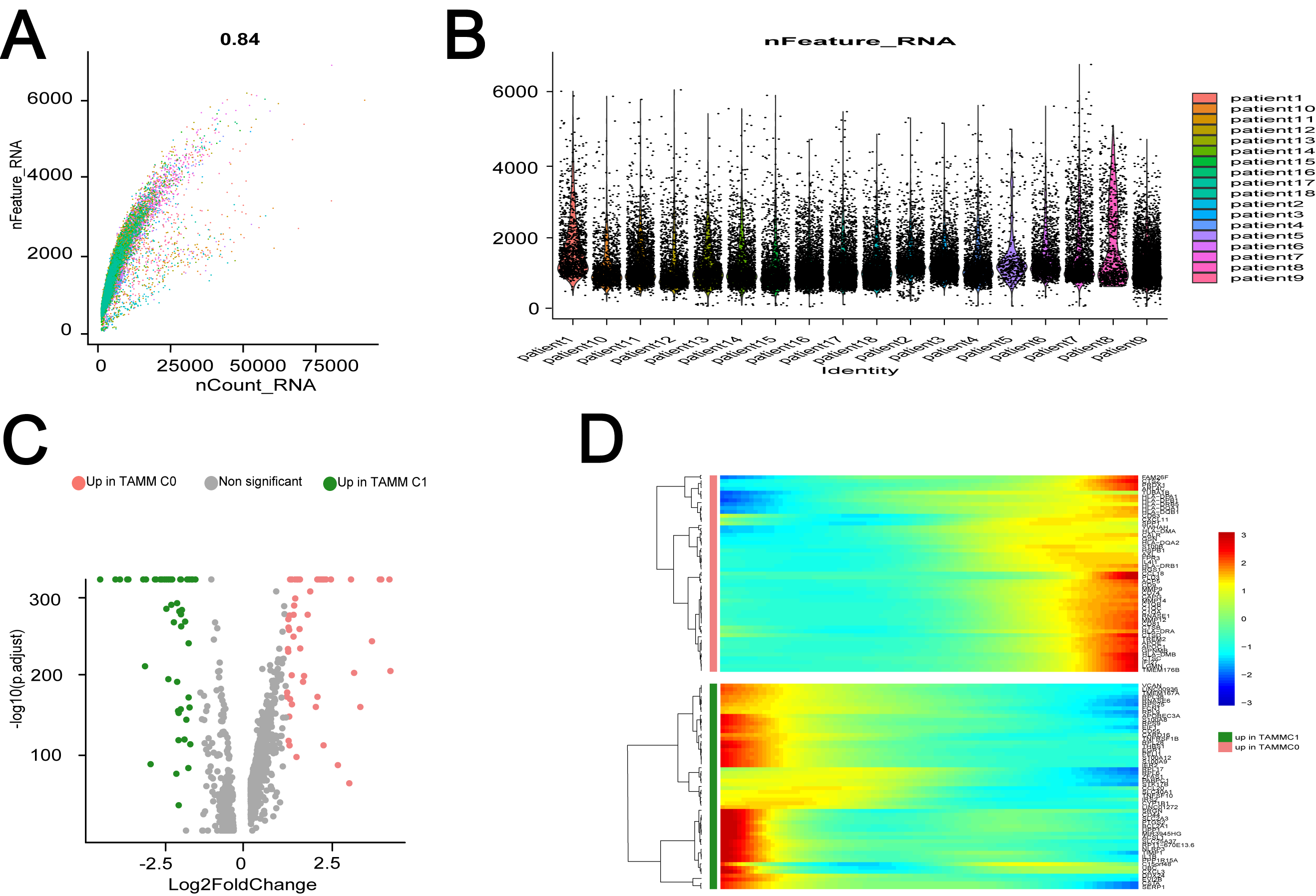

Supplement: Supplementary file 6 [file Image2.TIF]

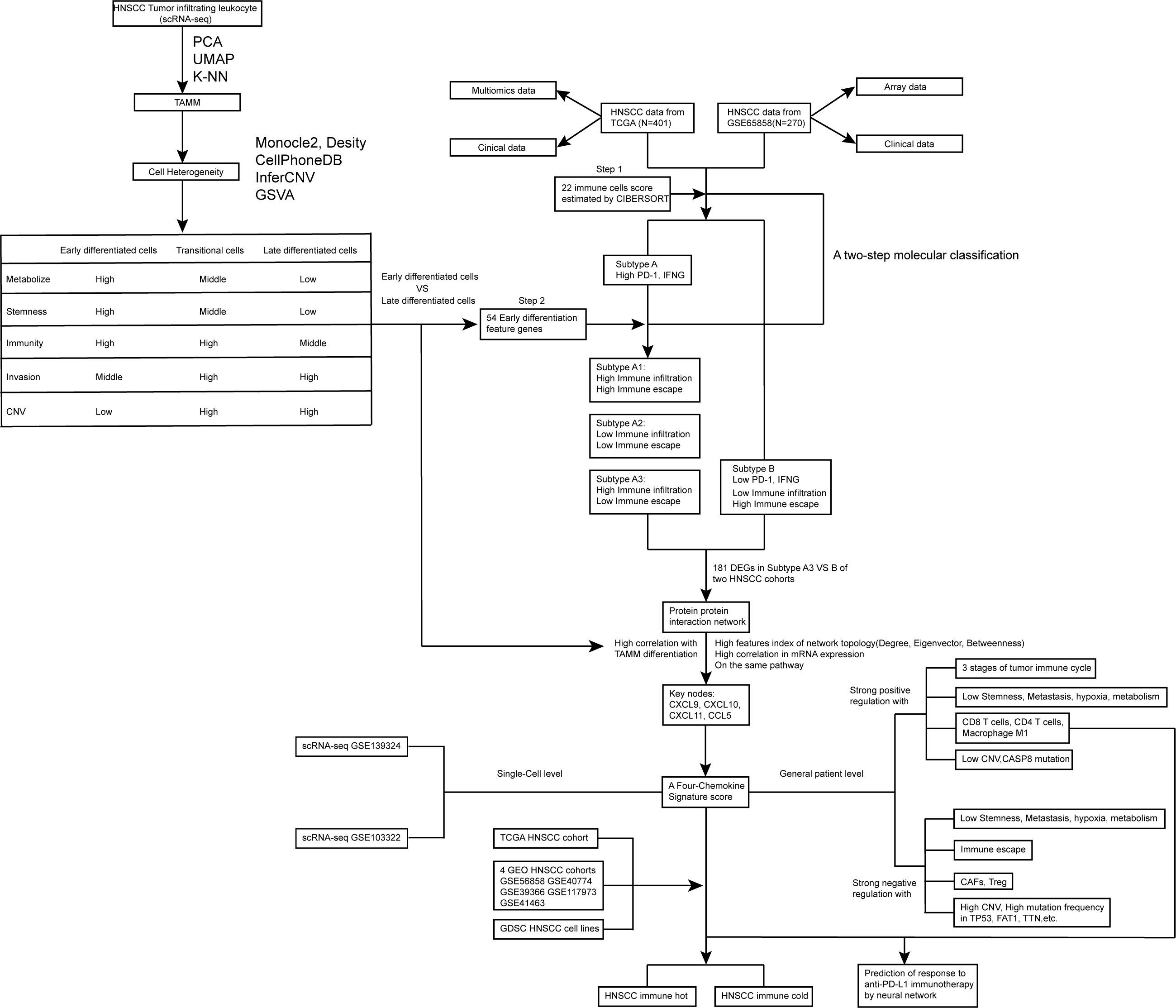

Supplement: Supplementary file 7 [file Image1.TIF]

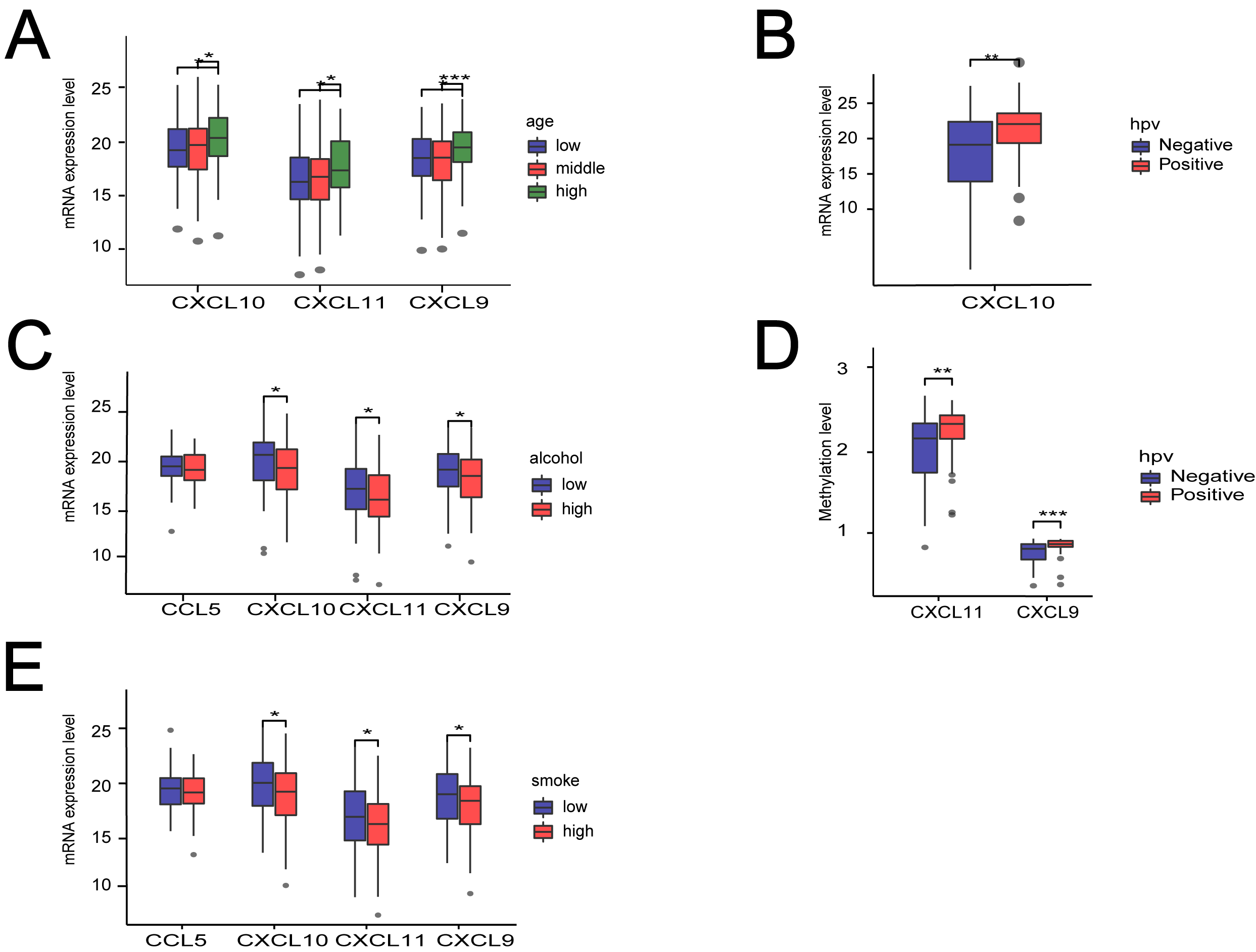

Supplement: Supplementary file 8 [file Image7.TIF]

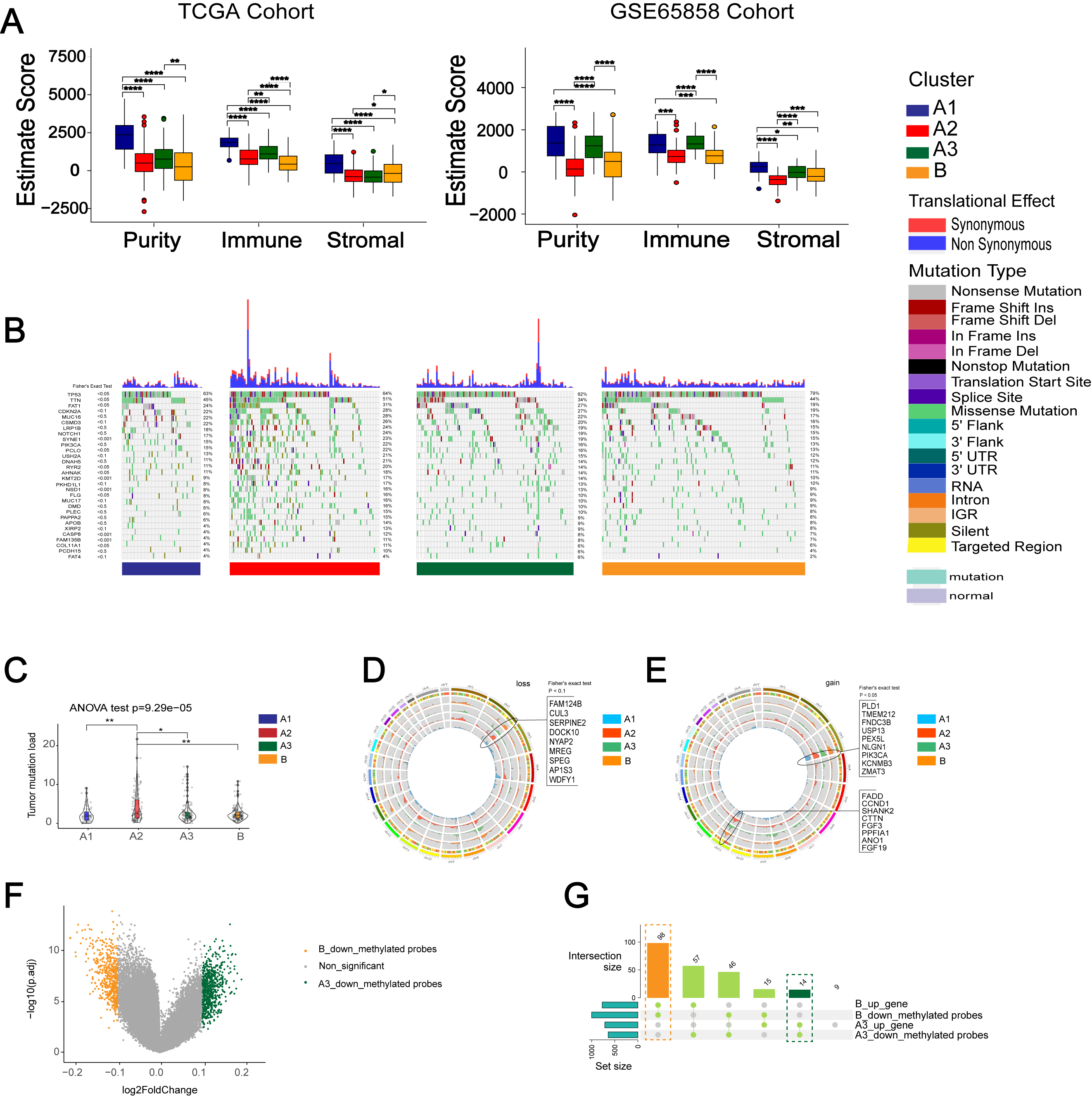

Supplement: Supplementary file 12 [file Image5.TIF]
